# Measurement-Efficient Few-Shot Vibration Fault Diagnosis via Physics-Informed Self-Supervised Learning and Adaptive Early Stopping

**DOI:** 10.3390/s26134252

**Published:** 2026-07-04

**Authors:** Zongzhe Ni, Xiancheng Ji, Jianjun Yi, Nuozhou Li, Hongxing Wang, Yifan Liu, Ying Yan

**Affiliations:** 1 School of Mechanical and Power Engineering, East China University of Science and Technology, Shanghai 200237, China; y15230007@mail.ecust.edu.cn (Z.N.);; 2 Robotics Engineering Center, The 21st Research Institute of CETC, Shanghai 200233, China

**Keywords:** few-shot fault diagnosis, self-supervised learning, data augmentation, deep reinforcement learning

## Abstract

Vibration-based fault diagnosis is widely used for rotating machinery health monitoring, but practical diagnosis is often limited by scarce fault labels and uncertain measurement length. Longer vibration records can improve decision reliability but increase sensing and computational cost, whereas overly short records may yield unreliable predictions under noise and measurement corruptions. This paper studies few-shot fault diagnosis as a measurement-constrained decision task, in which the model identifies the fault class and determines when sufficient vibration evidence has been acquired. We propose a measurement-efficient diagnosis framework that combines prior knowledge from unlabeled healthy signals, physically constrained augmentation of scarce labeled samples, and adaptive early stopping in a shared one-dimensional feature extractor. The framework is evaluated on the UORED-VAFCLS and Paderborn University bearing datasets under 6-, 8-, and 10-shot settings with controlled corruption levels. Results show robust diagnostic performance with fewer acquired vibration windows than with fixed-length inference. In the representative PU-Hard 8-shot setting, the proposed method achieves 80.26% accuracy with an average of 1.2432 acquired windows and reduces the evaluation cost J from 0.3929 to 0.2596 compared with fixed four-window inference. These results indicate that adaptive measurement improves the accuracy–cost trade-off in few-shot vibration diagnosis.

## 1. Introduction

### 1.1. Background

Rotating machinery is widely deployed in safety–critical industries such as wind power, railway transportation, and aero-engines. The operating condition of these systems is directly related to personnel safety, production continuity, and maintenance cost. Condition monitoring of rotating machinery relies on high-rate vibration measurements, where the decision latency and the amount of acquired data directly affect maintenance actions and computational load. In practice, the required measurement length is rarely known a priori: some sequences are informative enough within a short time, while others require longer measurements under noise and operating variability. Therefore, a diagnosis algorithm should be evaluated not only by accuracy, but also by its ability to reach a reliable decision with minimal measurement length. However, most existing diagnosis pipelines assume a pre-defined measurement duration (a fixed number of windows) and do not provide a principled stopping rule for deciding when sufficient evidence has been collected.

Traditional fault diagnosis methods mainly rely on signal-processing pipelines, expert-designed features and physically interpretable indicators [1,2,3,4]. Recent accelerometric-signal-based monitoring studies further show that vibration features and statistical indicators are useful beyond conventional bearing diagnosis. For example, Niola et al. [5] used accelerometric signal processing, feature selection, and a feedforward neural network to monitor torque/speed equilibrium points in an aircraft hybrid electric propulsion system. However, their performance degrades severely under complex operating conditions and strong noise, and they require substantial domain expertise to construct informative features and indicators.

Deep learning (DL)-based approaches can extract discriminative features in an end-to-end manner, but in industrial scenarios, they often suffer from label scarcity [6], class imbalance [7], and limited robustness under measurement variability [8].

Under such few-shot conditions, DL models are prone to learning incorrect decision boundaries or overfitting to specific working conditions.

Therefore, there is an urgent need for a measurement-efficient diagnosis framework that remains reliable under scarce labels and measurement variability, while being able to decide when enough measurement data have been acquired for a reliable judgment.

### 1.2. Related Work

Data augmentation. Both rule-based and generative data augmentation techniques have been widely used to enrich samples in few-shot scenarios [9]. Rule-based methods [10,11] focus on physically motivated perturbations in the time domain. For example, Overlap–Rotate–Jitter (ORJ) [12] combines overlapping segmentation, circular shifting, and additive noise to expand labeled data without changing the fault semantics, thereby improving robustness to noise. Generative augmentation based on generative adversarial networks (GANs) [13,14] and diffusion [15,16] models synthesizes minority-class samples in the time or time–frequency domain, and has shown clear improvements in highly imbalanced and extreme few-shot settings [17]. Representative methods such as balance-constrained conditional GANs (e.g., BCTGAN [18]) generate class-conditional signals to mitigate class bias, while other works embed adaptive generation and augmentation modules into end-to-end diagnosis networks and use time–frequency mappings as a bridge to improve robustness under operating variability. Rule-based perturbations are easy to control, but most of them modify only the time-domain waveform. They do not explicitly cover spectral attenuation, phase perturbation, or local information loss. Generative models provide a different way to increase diversity, but their training stability and physical consistency are still concerns in few-shot vibration diagnosis [19]. This motivates the use of bounded time–frequency perturbations rather than unconstrained sample generation [20].

Simulation, signal processing, and physics-informed hybrid diagnosis. Another important line of research combines physical simulation, signal processing, and learning-based classifiers. Sobie et al. [21] proposed a simulation-driven machine-learning framework for bearing race-fault classification, where bearing dynamic simulations are used to generate training data and the learned classifiers are validated on experimental datasets. This strategy reduces the dependence on historical in-service fault data, but its performance depends on the fidelity of the simulation model and the simulation-to-reality gap. Recent dynamic-modeling studies further show that bearing simulations can reproduce fault-frequency and modulation characteristics in complex rotating systems [22], providing physical guidance for feature design and model interpretation. In parallel, signal-processing–learning hybrids embed interpretable processing modules into neural networks. For example, classifier-guided neural blind deconvolution formulates blind deconvolution as a trainable denoising module and jointly optimizes periodic-impulse extraction and fault classification under noisy conditions [23]. Interpretable time–frequency deep models provide another related direction. Chen et al. [24] proposed a wavelet Kolmogorov–Arnold convolutional LSTM for spatial–temporal feature extraction and intelligent fault diagnosis, showing how wavelet-domain structure and interpretable network components can be combined for machinery diagnosis. Knowledge-informed deep networks also inject bearing-domain priors into the learning pipeline to improve diagnostic robustness [25]. More broadly, digital-twin-assisted diagnosis has become an active direction for linking simulation models, monitoring data and learning algorithms, although high-fidelity model construction, real-time diagnosis and simulation-to-reality generalization remain open challenges [26]. Recent cross-condition diagnosis studies further extend this line by using multi-source domain adaptation, universal domain adaptation, source-free adaptation, and federated adaptation to reduce the dependence on a fully labeled target domain [27,28,29]. These hybrid studies improve physical interpretability and data efficiency, but they usually focus on fixed-length fault classification. They rarely consider how many vibration windows should be acquired before making a decision under few-shot supervision.

Self-supervised learning and prior knowledge (SSL + Prior). Recent advances in self-supervised learning (SSL) show that by designing proxy tasks on unlabeled data, it is possible to learn reusable representations and greatly reduce the need for manual labels. For vibration signals, Tang et al. [30] proposed a prior-knowledge-enhanced time–frequency consistent SSL framework, where hand-crafted time-domain and frequency-domain features are used as pseudo-labels to supervise a dual-branch network [31]. This approach significantly improves few-shot diagnosis performance and strengthens the physical relevance of the learned representation. Meanwhile, time-series domain adaptation studies reveal the complementarity between time and frequency views: for example, Liu [32] demonstrates in unsupervised domain adaptation that frequency features are more discriminative whereas time-domain features are more adaptable across operating settings, and time–frequency collaborative learning [33] can further improve representation quality and class separability. These results indicate that injecting engineering priors into SSL proxy tasks [34,35,36,37,38,39] and interpretable time–frequency feature extractors is an effective way to reduce label dependency and encourage physically meaningful representations. These prior-driven SSL studies mainly use the learned representation for fixed-length classification. The connection between pretraining and downstream measurement decisions, especially when to stop acquiring windows, has received less attention.

Deep reinforcement learning (DRL) for diagnosis. Conventional DL-based diagnosis [40,41,42,43,44,45] typically adopts a fixed measurement length and performs static classification, which cannot answer the decision question of “how many windows are sufficient for a reliable judgment”. From a measurement perspective, this is a stopping-time problem closely related to classical sequential decision-making and sequential testing, where evidence is accumulated until a stopping criterion is met. To move beyond such static schemes, recent works [46,47] formulate fault diagnosis as an interactive sequential decision problem and employ deep reinforcement learning. Wang et al. [48] combined time–frequency representations with DRL for planetary gearbox diagnosis under varying operating conditions, showing that DRL can achieve more robust performance than static DL models in dynamic and noisy environments. In few-shot settings, DRL has also been used for adaptive prototype refinement, such as the “Learn to Supervise” framework [49] that refines few-shot prototypes via RL to enhance motor fault recognition. For compound faults and imbalanced data, improved Deep Q-Network (DQN) [50] variants have been developed for multi-label diagnosis and class-aware policy learning, highlighting the importance of reward design and policy constraints in industrial diagnosis. More recently, convolution–Transformer hybrid agents with DRL, such as the Convolutional Transformer Q-Network (CTQN) [51] with double-priority experience replay (LiteDPER), have achieved faster convergence and higher accuracy and provide analyzable Q-value distributions. Despite these advances, most DRL-based methods still rely on fixed input length and do not explicitly optimize the joint trade-off between diagnostic accuracy and decision efficiency.

Existing few-shot fault diagnosis studies mainly focus on improving classification accuracy under scarce labels. Prior-guided SSL methods improve representation learning, augmentation methods increase sample diversity, and DRL-based diagnosis methods introduce sequential decision-making. However, these directions are usually studied separately, and most methods still assume a fixed input length during inference. This work focuses on their system-level coupling through a shared backbone for measurement-constrained few-shot diagnosis, where the model must decide both what the fault is and when enough vibration windows have been acquired.

### 1.3. Research Gaps

The literature above leaves four issues that are relevant to measurement-efficient few-shot diagnosis.

First, augmentation for vibration signals still has limited coverage of measurement distortions. Simple ORJ-type operations mainly act in the time domain, whereas practical measurements may also contain spectral attenuation, phase drift, local signal loss, and mild time-warping. Generative augmentation can increase diversity, but its training stability and physical consistency are difficult to guarantee when only a few labeled fault samples are available.

Second, simulation-driven and signal-processing–learning hybrid methods improve data efficiency and physical interpretability, but they are usually designed for fixed-length fault classification. Simulation-trained classifiers depend on the fidelity of the bearing dynamic model, and signal-processing hybrids often focus on extracting more discriminative features from a pre-defined signal segment. These studies do not directly address the online decision problem of how many measurement windows should be acquired before diagnosis.

Third, prior-driven SSL has mostly been used to initialize a feature extractor for fixed-length classification. Time-domain and frequency-domain statistics can provide useful vibration priors, but the learned representation is rarely reused for an online stopping policy. This leaves a gap between prior-informed representation learning and measurement-length control.

Fourth, most diagnosis pipelines still use a pre-defined input length during inference. From a measurement viewpoint, this fixes the acquisition cost before the model has seen the sample. A more suitable formulation is to let the model decide whether the current evidence is sufficient or another window should be acquired.

From this viewpoint, few-shot diagnosis is treated as a measurement-constrained stopping problem. The target is not only to improve classification accuracy, but also to reduce unnecessary vibration-window acquisition under scarce labels and controlled measurement corruptions.

### 1.4. Contributions

This paper studies few-shot vibration diagnosis with an explicit measurement budget. The contribution is the coupling of prior-guided pretraining, physically constrained support-set augmentation, and adaptive stopping within the same 1D backbone.

The main contributions are as follows:

1. Few-shot vibration diagnosis is formulated as a measurement-constrained stopping problem. The model predicts the fault class and decides whether the current window sequence contains enough evidence for a final decision.
2. PK-SSL, PhORJ and ESDRL are connected through a shared backbone. PK-SSL initializes the feature extractor from unlabeled healthy signals, PhORJ augments the scarce labeled support set, and ESDRL uses the resulting feature and confidence trajectory for STOP/CONTINUE decisions.
3. The stopping policy is trained with a finite-horizon MDP. Its state contains the current feature, a history-aggregated feature, the classifier confidence, and the normalized step index, allowing the policy to trade terminal correctness against measurement cost.
4. Experiments are conducted on UORED-VAFCLS and PU under representative few-shot settings with K=6/8/10 labeled samples per class. The evaluation includes controlled measurement corruptions, baseline fairness checks, repeated evaluation-subset tests, calibration analysis, fixed-window and confidence-threshold comparisons, stopping-step statistics, computational-cost analysis, and module ablations.

The remainder of this paper is organized as follows. Section 2 presents the proposed methodology, including PK-SSL, PhORJ, the shared 1D backbone, and ESDRL. Section 3 describes the datasets, experimental protocols, baseline comparisons, efficiency analysis, and ablation studies. Section 4 concludes the paper and discusses future work.

## 2. Method

### 2.1. Problem Formulation and Overall Framework

In this work, we consider a few-shot fault diagnosis scenario, where each vibration signal is represented as

(1) $$x\in R^{L},$$

with *L* denoting the signal length.

We are given a set of unlabeled samples

(2) $$D_{u}={\left\{x_{i}^{\left(u\right)}\right\}}_{i=1}^{N_{u}},$$

and a labeled few-shot set

(3) $$D_{l}={\left\{(x_{j}^{\left(l\right)},y_{j})\right\}}_{j=1}^{N_{l}},\;y_{j}\in \left\{1,2,\ldots ,C\right\},$$

where *C* is the number of fault classes. The labeled set $D_{l}$ follows a *C*-way *K*-shot few-shot protocol. In the experiments, we focus on representative few-shot settings with K=6/8/10 labeled samples per class. These settings emulate engineering diagnosis scenarios where only a small number of labeled samples are available for each fault category. Our goal is to learn a diagnosis policy that, under limited labeled data, achieves high classification accuracy while using as few time windows as possible for online decision-making.

To emulate online monitoring, the raw sequence x is segmented into a series of fixed-length windows with length $L_{w}$ and hop size $L_{s}$:

(4) $$x\;⟶\;\left\{x^{\left(1\right)},\;x^{\left(2\right)},\;\ldots ,\;x^{\left(T_{\max}\right)}\right\},$$

where $T_{\max}$ denotes the maximum number of windows allowed to be acquired.

At each time step *t*, the current window $x^{\left(t\right)}$ is fed into the shared 1D multi-scale attention backbone to obtain a feature vector

(5) $$h_{t}=g_{\theta_{b}}\left(x^{\left(t\right)}\right),$$

where $\theta_{b}$ denotes the parameters of the shared backbone encoder and a linear classifier produces the class prediction and its confidence:

(6) $$p_{t}=\mathrm{softmax}\left(W_{c}h_{t}+b_{c}\right),\;{\hat{y}}_{t}=\arg\max_{k}p_{t,k},\;{\mathrm{conf}}_{t}=\max_{k}p_{t,k}.$$

where $p_{t,k}$ is the predicted probability that window *t* belongs to class *k*. Because this confidence score is used by the stopping policy, its calibration is examined in Section 3.4.4.

Based on this, we formulate early-stopping diagnosis as a finite-horizon MDP defined on the sliding-window sequence. Unlike static schemes that always use a fixed number of windows, the MDP formulation explicitly models the trade-off between information gain and measurement cost at each step. At each step *t*, the agent observes a state composed of the current feature, a history-aggregated feature, the diagnosis confidence, and the step index. We define the running-mean feature as

(7) $${\bar{h}}_{t}=\frac{1}{t}\sum_{\tau =1}^{t}h_{\tau },$$

and construct the DRL state as

(8) $$s_{t}=\left[\;h_{t},\;{\bar{h}}_{t},\;{\mathrm{conf}}_{t},\;t/T_{\max}\;\right].$$

The agent then selects an action

(9) $$a_{t}\in \left\{\mathrm{CONTINUE},\;\mathrm{STOP}\right\}.$$

If $a_{t}=\mathrm{CONTINUE}$, the system reads the next window $x^{\left(t+1\right)}$ and updates the state; if $a_{t}=\mathrm{STOP}$, the current prediction ${\hat{y}}_{t}$ is output as the final diagnosis result and the episode terminates. By carefully designing a process reward and a terminal reward, the agent is explicitly encouraged to obtain a reliable decision with the minimum possible number of windows.

The design is guided by the following dependency among the modules. Under few-shot supervision, the backbone first needs a stable initialization; this motivates PK-SSL on unlabeled healthy signals. The labeled support set is then too small to cover realistic measurement variability; this motivates PhORJ to enlarge the support distribution with physically constrained perturbations. Finally, online diagnosis should not always use a fixed number of windows; this motivates ESDRL to use the learned representation and confidence trajectory for adaptive stopping. These stages are connected through the shared backbone and the common accuracy–measurement–cost objective.

**Figure 1:**
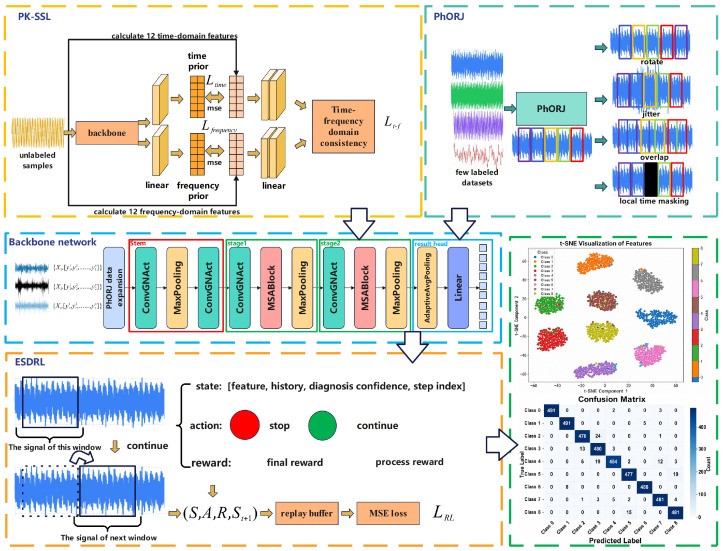
Overall framework of the proposed measurement-efficient few-shot diagnosis system. PK-SSL, PhORJ, and ESDRL are coupled through a shared 1D backbone, so that representation learning, augmentation, and stopping decisions support the same accuracy–measurement–cost objective.

As illustrated in Figure 1, the proposed method is an end-to-end framework composed of the following four modules:

**PK-SSL pretraining module.** This module exploits a large amount of unlabeled vibration data to pretrain the backbone network using 12 time-domain + 12 frequency-domain handcrafted statistics as pseudo-labels together with a time–frequency consistency constraint. Only unlabeled data are used in this stage; fault labels are not involved, and the objective is purely to learn a physically meaningful initialization of the backbone representation. In other words, PK-SSL performs a one-step self-supervised pretraining so that the backbone can capture prior knowledge from unlabeled signals.

**PhORJ data augmentation module.** For the labeled few-shot data, PhORJ extends the classical ORJ (Overlap–Rotate–Jitter) scheme by injecting physics-constrained perturbations in both the time and frequency domains, including band-wise masking and boosting, phase jitter, and local time masking. These operations generate diverse but physically plausible training samples, improving intra-class diversity and robustness to noise for the labeled set. The unlabeled signals used for PK-SSL are not augmented by PhORJ; PhORJ is applied only to the labeled support samples.

**One-dimensional multi-scale backbone.** This is the shared backbone $g_{\theta_{b}}\left(\cdot \right)$ pretrained by PK-SSL and further fine-tuned in the supervised and DRL stages. The backbone consists of a stem block, two multi-scale convolutional stages, and a final result head, as shown in Figure 1. The stem adopts a Conv1D + GroupNorm + SiLU + MaxPooling stack to roughly compress the temporal length and suppress noise. Each multi-scale stage is built by parallel depthwise separable convolutions with different kernel sizes and dilation rates, followed by SE channel attention, so as to capture fault patterns at different temporal scales. The result head applies global adaptive average pooling and a linear layer to produce a 256-dimensional feature. On the one hand, these features are used for visualization and analysis via t-SNE and confusion matrices; on the other hand, the feature vector $h_{t}$ concatenated with the classification output forms the state input for the DRL agent.

**DRL-based early-stopping diagnosis (ESDRL).** The RL module takes the backbone feature and classifier output as state $s_{t}$, and outputs an action $a_{t}\in \left\{\mathrm{STOP},\mathrm{CONTINUE}\right\}$. During training, transition tuples $\left(s_{t},a_{t},r_{t},s_{t+1}\right)$ are stored in a replay buffer, and a Double-DQN-style algorithm is used to minimize the DRL loss $L_{\mathrm{RL}}$. In this way, the agent gradually learns a policy that achieves a favorable trade-off between diagnostic accuracy and the number of acquired windows.

Figure 1 summarizes the formal problem definition of few-shot early-stopping fault diagnosis and the end-to-end workflow of these four modules: PK-SSL first pretrains the backbone on unlabeled data; PhORJ then augments the labeled few-shot set; the 1D multi-scale backbone and ESDRL module are finally jointly optimized to realize “early yet reliable” diagnosis. This subsection provides the overall formulation and framework; the following Section 2.2, Section 2.3, Section 2.4 and Section 2.5 describe in detail the structures and loss functions of the PK-SSL pretraining, PhORJ data augmentation, 1D backbone, and ESDRL early-stopping strategy, respectively.

### 2.2. Prior-Knowledge-Driven Time–Frequency Consistent SSL

To obtain physically meaningful initial representations under few-shot conditions, we first perform prior-knowledge-driven self-supervised pretraining on the unlabeled dataset $D_{u}$. The core idea is to use time-domain and frequency-domain statistics as pseudo-labels and to enforce consistency between the two views, so that the backbone can learn prior-informed time–frequency representations without any manual fault labels.

#### 2.2.1. Time–Frequency Prior Features

Given an unlabeled vibration sequence $x\in R^{L}$, we segment it into fixed-length windows according to the sliding scheme in Section 2.1:

(10) $${\left\{x^{\left(t\right)}\right\}}_{t=1}^{T_{\max}}.$$

For each window, we describe its prior knowledge using a set of hand-crafted statistics. Specifically, we adopt 12 time-domain and 12 frequency-domain indicators that are widely used as health indices in vibration-based bearing diagnosis, covering dispersion, impulsiveness, and spectral distribution. These statistics can be computed reliably from short windows and provide a compact, physically meaningful description of each window.

For clarity and reproducibility, their exact definitions are listed in Table 1 and Table 2, where *N* is the number of samples in a window and 1(·) denotes the indicator function. Let X(k) denote the complex coefficient of the one-sided spectrum (i.e., the output of a real-input FFT, rFFT, for real-valued signals) and |X(k)| its magnitude; let *K* be the number of frequency bins; and let $f_{k}$ be the frequency of the *k*-th bin with $f_{\max}=\max_{k}f_{k}$. The sets $B_{1},\ldots ,B_{4}$ partition the bins into four equal-width frequency bands with $K_{i}=\left|B_{i}\right|$, and $B_{\mathrm{high}}$ denotes the predefined high-frequency band (with $K_{h}=\left|B_{\mathrm{high}}\right|$).

(11) $$t^{\left(t\right)}={\left[T_{1},\ldots ,T_{12}\right]}^{⊤},\;f^{\left(t\right)}={\left[F_{1},\ldots ,F_{12}\right]}^{⊤},$$

where $T_{i}$ and $F_{i}$, respectively, denote the *i*-th time-domain and frequency-domain prior indicators.

#### 2.2.2. Dual-Head Regression Structure

As shown in Figure 1, PK-SSL employs a shared 1D multi-scale backbone encoder $g_{\theta_{b}}\left(\cdot \right)$ (the same backbone as in Section 2.4, whose weights are reused in the downstream few-shot and DRL stages). For each window $x^{\left(t\right)}$, we feed the time-domain window into the backbone and obtain a shared representation:

(12) $$h^{\left(t\right)}=g_{\theta_{b}}\;\left(x^{\left(t\right)}\right).$$

The time-domain and frequency-domain pseudo-labels are computed from the same window $x^{\left(t\right)}$ according to Table 1 and Table 2, where the frequency-domain indicators are extracted from the one-sided rFFT magnitude spectrum $|X^{\left(t\right)}\left(k\right)|$ (with K=N/2+1 bins for a length-*N* window). Note that the spectrum is not used as an additional backbone input; it is only used to construct frequency-domain pseudo-labels.

On top of $h^{\left(t\right)}$, we attach two lightweight regression heads, one for predicting time-domain indicators and the other for predicting frequency-domain indicators:

(13) $${\hat{t}}^{\left(t\right)}=r_{\theta_{t}}\;\left(h^{\left(t\right)}\right),\;{\hat{f}}^{\left(t\right)}=r_{\theta_{f}}\;\left(h^{\left(t\right)}\right),$$

where ${\hat{t}}^{\left(t\right)}$ and ${\hat{f}}^{\left(t\right)}$ denote the network predictions of the time-domain and frequency-domain pseudo-labels, respectively.

One training objective is to minimize the mean squared error (MSE) between predicted and target statistics over all unlabeled samples and windows:

(14) $$L_{\mathrm{time}}=\frac{1}{N_{u}T_{\max}}\sum_{i,t}∥{\hat{t}}_{i}^{\left(t\right)}-t_{i}^{\left(t\right)}{∥}_{2}^{2},$$

(15) $$L_{\mathrm{freq}}=\frac{1}{N_{u}T_{\max}}\sum_{i,t}∥{\hat{f}}_{i}^{\left(t\right)}-f_{i}^{\left(t\right)}{∥}_{2}^{2},$$

where the subscript *i* indexes unlabeled samples and *t* indexes windows within each sequence.

#### 2.2.3. Time–Frequency Consistency Regularization and Total SSL Loss

Regressing pseudo-labels alone encourages the model to fit the time-domain and frequency-domain statistics via two separate heads, but does not explicitly enforce time–frequency coherence.

Since the two pseudo-label vectors ${\hat{t}}^{\left(t\right)}$ and ${\hat{f}}^{\left(t\right)}$ consist of heterogeneous indicators with different physical meanings and scales, we introduce a lightweight learnable time–frequency alignment projector A to align them.

Specifically, $A\in R^{12\times 12}$ is implemented as a linear layer and is optimized jointly with the backbone and the two regression heads. The time–frequency consistency loss is defined as:

(16) $$L_{tf}=\frac{1}{N_{u}T_{\max}}\sum_{i,t}\frac{1}{2}{∥A\;{\hat{t}}_{i}^{\left(t\right)}-{\hat{f}}_{i}^{\left(t\right)}∥}_{2}^{2}.$$

Combining the above terms, the overall loss of the PK-SSL pretraining stage is

(17) $$L_{SSL}=\lambda_{t}L_{\mathrm{time}}+\lambda_{f}L_{\mathrm{freq}}+\lambda_{tf}L_{tf},$$

where $\lambda_{t}$, $\lambda_{f}$ and $\lambda_{tf}$ balance the contributions of the three supervision signals. In all experiments, we fix $\lambda_{t}=\lambda_{f}=1.0$ and $\lambda_{tf}=0.1$, so that the time- and frequency-domain regression losses contribute equally, while the time–frequency consistency loss acts as a weaker regularization term.

The role of the consistency term is further examined in the ablation study. The “Time + Freq w/o Consistency” variant removes the $\lambda_{tf}L_{tf}$ term, while the full PK-SSL keeps the default $\lambda_{tf}=0.1$.

During PK-SSL pretraining, the backbone parameters $\theta_{b}$, the regression-head parameters, and the time–frequency alignment projector parameters A are updated jointly. After pretraining, the two regression heads and the projector A are discarded, and we only retain the backbone parameters $\theta_{b}$ as the initialisation for downstream tasks.

### 2.3. Physics-Constrained ORJ

Under few-shot conditions, relying only on a small number of labeled samples is insufficient to cover the diverse noise patterns and local mismatches that may occur in real measurements. To enrich the diversity and robustness of supervised training data, while avoiding the potential physical distortions introduced by generative models, we build a PhORJ module on top of the classical ORJ scheme. PhORJ introduces physically meaningful perturbations in both the time and frequency domains. It is only applied to the labeled few-shot set $D_{l}$, whereas the unlabeled dataset $D_{u}$ used in PK-SSL remains unchanged.

Let the original labeled samples be $\left(x_{i},y_{i}\right)$, where $x_{i}\in R^{L}$ is a vibration sequence of length L=4096, and $y_{i}$ is its fault label. PhORJ defines a family of physically motivated transformation operators $A={\left\{A_{k}\right\}}_{k=1}^{K}$. At each augmentation step, one or several operators are randomly sampled and composed to generate an augmented sample; each $A_{k}$ corresponds to a specific augmentation or a combination of augmentations (e.g., rotation + overlap recompose). For each original sample $x_{i}$, we randomly sample *M* operators from A and apply them to obtain

(18) $${\tilde{x}}_{i}^{\left(m\right)}=A_{k_{m}}\left(x_{i}\right),\;m=1,\ldots ,M,$$

while keeping the labels unchanged

(19) $${\tilde{y}}_{i}^{\left(m\right)}=y_{i}.$$

Finally, we obtain the enlarged training set

(20) $$D_{l}^{\mathrm{aug}}={\left\{(x_{i},y_{i})\right\}}_{i=1}^{N_{l}}\;\cup \;\left\{({\tilde{x}}_{i}^{\left(m\right)},y_{i})\right\}.$$

#### 2.3.1. Basic ORJ Operations

The classical ORJ scheme uses three simple yet effective time-domain perturbations to mimic changes in sampling origin and noise level without altering the underlying fault semantics.

Rotate (circular shift). To simulate different rotor phases or sampling origins, the signal is circularly shifted. Given a maximum shift $\Delta_{\max}$, we randomly draw $\delta ∼U[-\Delta_{\max},\Delta_{\max}]$ and obtain

(21) $$x_{\mathrm{rot}}\left[n\right]=x\left[\left(n+\delta \right)\;\mathrm{mod}\;L\right],$$

where U[·,·] denotes a discrete uniform distribution over the corresponding integer range, and *L* is the signal length. This perturbation changes the sampling origin while leaving the waveform shape unchanged. In our implementation, the maximum shift is set to $\Delta_{\max}=256$ samples, and the shift δ is sampled uniformly from U{−256,…,256}.

Jitter (additive noise). To simulate measurement noise and slight operating condition fluctuations, we add zero-mean Gaussian noise with a standard deviation proportional to the original signal:

(22) $$x_{\mathrm{jit}}\left[n\right]=x\left[n\right]+\sigma e_{n},\;e_{n}∼N\left(0,1\right),\;\sigma =\alpha \;\mathrm{std}\left(x\right),$$

where α=0.02 controls the noise level.

Overlap–recompose. To model local misalignment and limited observation windows, we construct an overlap–add recompose with *P* windowed segments. Let the *p*-th segment be extracted around the anchor start (p−1)H with a small random offset $\delta_{p}$:

(23) $$x_{p}\left[\tau \right]=x\left(\left(p-1\right)H+\tau +\delta_{p}\;\;\mathrm{mod}\;\;L\right),\;\tau =0,\ldots ,W-1,$$

where *H* is the hop size, *W* is the window length, and $\delta_{p}$ is an integer offset. We then reconstruct the signal by overlap–add with window weighting and weight normalization:

(24) $$\tilde{x}\left[n\right]=\frac{\sum_{p=1}^{P}w\left[\tau \right]\;x_{p}\left[\tau \right]\;1\left(n=\left(p-1\right)H+\tau \right)}{\sum_{p=1}^{P}w\left[\tau \right]\;1\left(n=\left(p-1\right)H+\tau \right)}.$$

In our implementation, we set P=4, W=1024 and H=512, apply a Hann window w(·), and sample $\delta_{p}∼U\left\{-128,\ldots ,128\right\}$. Samples not covered by any window keep their original values.

#### 2.3.2. Frequency- and Time-Domain Physical Augmentations

To further account for more complex real-world effects such as band mismatch, phase perturbation, and local information loss, we design four additional augmentation operators on top of ORJ.

Frequency-band masking and boosting. Given a real-valued signal *x*, we compute its one-sided complex spectrum X=rFFT(x) using the real-input FFT (rFFT). On the magnitude spectrum |X|, we randomly select one or several narrow-band intervals [a,b] and perform

(25) |X[k]|←g|X[k]|,k∈[a,b],

where g∈[0.5,0.9] corresponds to attenuation and g∈[1.1,1.5] to boosting. This operation enhances or suppresses local spectral peaks. The augmented signal is then reconstructed by the inverse real FFT (irFFT):

(26) $$x_{\mathrm{fb}}=\mathrm{irFFT}\;\left(\left|X\right|e^{j∠X}\right).$$

Phase jitter in the frequency domain. To simulate phase drifts caused by speed fluctuation or local stiffness changes, we add small Gaussian perturbations to the phase spectrum while keeping the magnitude unchanged:

(27) $$∠X\left[k\right]\leftarrow ∠X\left[k\right]+\eta_{k},\;\eta_{k}∼N\left(0,\sigma_{\phi }^{2}\right),$$

and reconstruct the signal as

(28) $$x_{\mathrm{pj}}=\mathrm{irFFT}\;\left(\left|X\right|e^{j∠X^{\prime }}\right),$$

where $∠X^{\prime }$ denotes the perturbed phase spectrum. In the experiment, we set $\sigma_{\phi }=0.12$ for the phase-jitter operator.

Global time-warp. To emulate slight speed-up or slow-down effects, we uniformly stretch or compress the time axis by a factor s∈[1−γ,1+γ], followed by linear interpolation back to length *L*:

(29) $$x_{\mathrm{tw}}\left[n\right]=x\;\left(\frac{n}{s}\right),\;\gamma \approx 0.02.$$

Local time masking. To mimic short-time sensor drop-out or local impacts, we randomly choose one time segment of length $\omega \in [\omega_{\min},\omega_{\max}]$, denoted by M, and apply

(30) $$x_{\mathrm{tm}}\left[n\right]=\left\{\begin{array}{cc} x[n], & n\notin M,\\ x\left[n_{\ell }\right]+\frac{n-n_{\ell }}{n_{r}-n_{\ell }}\left(x\left[n_{r}\right]-x\left[n_{\ell }\right]\right), & n\in M, \end{array}\right.$$

where $n_{\ell }$ and $n_{r}$ are the left and right boundary indices adjacent to the masked segment. The above operators can be randomly combined, e.g., “band boosting + phase jitter + rotation + overlap-recompose + jitter noise”, to increase the time–frequency diversity of few-shot training samples while preserving physical plausibility and fault semantics.

### 2.4. One-Dimensional Multi-Scale Attention Backbone

**Figure 2:**
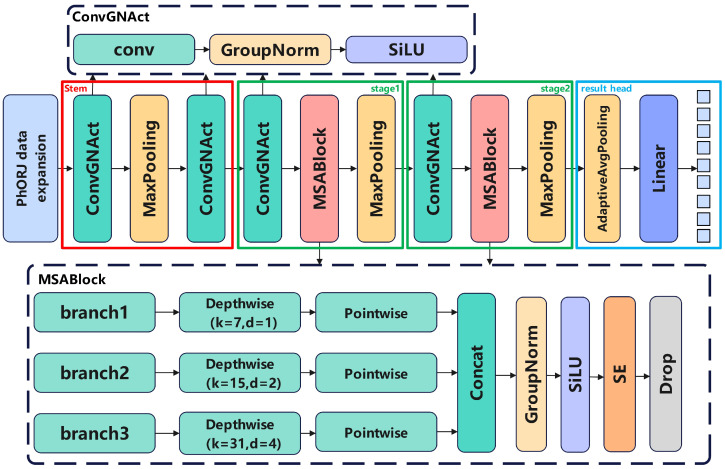
Architecture of the 1D multi-scale attention backbone. The parallel convolutional branches capture vibration patterns at different temporal scales, and the resulting feature vector is shared by PK-SSL pretraining, few-shot classification, and ESDRL stopping decisions.

As illustrated in Figure 2, we adopt a one-dimensional multi-scale attention backbone $g_{\theta_{b}}\left(\cdot \right)$ as a unified feature extractor throughout PK-SSL pretraining and the subsequent reinforcement learning stages. For any vibration window $x^{\left(t\right)}\in R^{1\times L_{w}}$ of length $L_{w}$, the backbone outputs a *d*-dimensional feature vector

(31) $$h_{t}=g_{\theta_{b}}\;\left(x^{\left(t\right)}\right)\in R^{d}.$$

In this work, we set d=256. The same feature representation is used to regress time-/frequency-domain pseudo-labels in PK-SSL and later serves as the input to both few-shot classification and DRL-based early-stopping decision-making.

The backbone network consists of a stem convolutional block, two multi-scale convolutional stages, and a head block:

Stem block. The stem block uses a 1D convolution followed by Group Normalization and a non-linear activation, and then a max-pooling layer. It performs initial temporal down-sampling and suppresses high-frequency noise.

Multi-scale convolutional stages. Each multi-scale convolutional stage consists of three parallel depthwise convolution branches with different receptive fields (e.g., kernel sizes 7/15/31 capturing short-, medium-, and long-term periodic components). The outputs of the three branches are concatenated along the channel dimension and then fed into an SE channel-attention block, which adaptively emphasizes fault-sensitive channels.

Head block. The head block applies global adaptive average pooling to remove the temporal dimension, followed by a linear projection to obtain the final 256-dimensional feature vector.

Benefiting from 1D convolutions, depthwise separable convolutions and GroupNorm, the proposed backbone is computationally efficient and remains stable under small-batch training, which is crucial in few-shot scenarios.

### 2.5. DRL-Based Early-Stopping Diagnosis (ESDRL)

In Section 2.1, we have formulated few-shot fault diagnosis as a sliding-window finite-horizon MDP. In this section, we instantiate the ESDRL module by specifying the state and action space, the reward design, and the learning objective, and explain how it collaborates with the previously introduced backbone $g_{\theta_{b}}\left(\cdot \right)$.

#### 2.5.1. State and Action Space

As defined in Section 2.1, at each step *t*, the backbone extracts the feature vector $h_{t}$, and the classifier produces the probability, prediction, and confidence $p_{t}$, ${\hat{y}}_{t}$, and ${\mathrm{conf}}_{t}$ (see Equations (6)–(9)). The DRL state is constructed as $s_{t}$ in Equation (8), and the action space is A={CONTINUE,STOP} as in Equation (9).

If the agent chooses CONTINUE and $t<T_{\max}$, the environment reveals the next window $x^{\left(t+1\right)}$ and the agent observes $s_{t+1}$; otherwise, the episode terminates and the current prediction ${\hat{y}}_{T}$ is taken as the final diagnostic result.

The goal of ESDRL is therefore to guarantee reliable classification accuracy while completing the diagnosis with as few windows as possible.

#### 2.5.2. Reward Design

To balance accuracy and efficiency, the reward function is decomposed into a process reward and a terminal reward.

Process reward $r_{t}^{\mathrm{proc}}$ (for $a_{t}=\mathrm{CONTINUE}$). Intuitively, we hope that each additional window contributes to a genuine increase in confidence; otherwise, the agent should be discouraged from acquiring too many windows. For step t<T with action $a_{t}=\mathrm{CONTINUE}$ we define

(32) $$r_{t}^{\mathrm{proc}}=\max\left(0,\;{\mathrm{conf}}_{t+1}-{\mathrm{conf}}_{t}\right)-c_{\mathrm{step}},$$

where $c_{\mathrm{step}}>0$ is the per-step cost used in the ESDRL reward. The selected value is $c_{\mathrm{step}}=0.05$, and it is fixed for all test-set experiments. Thus, a CONTINUE action is penalized unless the next-window confidence gain is large enough to compensate for this step cost.

Terminal reward $r_{T}^{\mathrm{final}}$ (at the terminal step *T*). At termination, the reward depends on whether the final prediction is correct:

(33) $$r_{T}^{\mathrm{final}}=\left\{\begin{array}{cc} +1, & {\hat{y}}_{T}=y,\\ -1, & {\hat{y}}_{T}\neq y, \end{array}\right.$$

The overall return of an episode is defined as

(34) $$R=\sum_{t=1}^{T}\gamma^{t-1}r_{t},\;r_{t}=\left\{\begin{array}{cc} r_{t}^{\mathrm{proc}}, & t<T,\\ r_{T}^{\mathrm{final}}, & t=T, \end{array}\right.$$

where γ∈(0,1] is the discount factor. Based on the validation results, γ was set to 0.90. The terminal step *T* is the step at which the agent chooses STOP, or $T=T_{\max}$ if the agent never stops early.

#### 2.5.3. Q-Network and Joint Loss

During ESDRL training, we jointly optimize the supervised classifier head $\theta_{c}=\left\{W_{c},b_{c}\right\}$, the Q-network parameters $\theta_{q}$, and the shared backbone encoder parameters $\theta_{b}$. The Q-network takes $s_{t}=\left[h_{t},{\bar{h}}_{t},{\mathrm{conf}}_{t},t/T_{\max}\right]$ as input, where $h_{t}=g_{\theta_{b}}\left(x^{\left(t\right)}\right)$ and ${\bar{h}}_{t}$ is the running-mean feature defined in Equation (7). This simple history summary mitigates myopic decisions by allowing the policy to accumulate evidence across observed windows. Therefore, gradients from the DRL loss $L_{\mathrm{RL}}$ also backpropagate to $\theta_{b}$ through $h_{t}$. The target network parameters $\phi_{q}$ are updated by soft updates from $\theta_{q}$ at each optimization step with factor τ.

We employ a standard deep Q-network $Q_{\theta_{q}}\left(s,a\right)$ to approximate the optimal state–action value function and stabilize training with Double-DQN and an experience replay buffer. For each sampled transition $\left(s_{t},a_{t},r_{t},s_{t+1}\right)$, the target Q-value is computed as

(35) $$y_{t}=\left\{\begin{array}{cc} r_{t}+\gamma \;Q_{\phi_{q}}\;\left(s_{t+1},\arg\max_{a^{\prime }}Q_{\theta_{q}}\left(s_{t+1},a^{\prime }\right)\right),\; & \mathrm{non-terminal},\\ r_{t}, & \mathrm{terminal}, \end{array}\right.$$

where $\phi_{q}$ denotes the parameters of the target network. The DRL loss is the average Huber loss between the target and predicted Q-values:

(36) $$L_{\mathrm{RL}}=\frac{1}{B}\sum_{t=1}^{B}\rho \left(y_{t}-Q_{\theta_{q}}\left(s_{t},a_{t}\right)\right),$$

where *B* is the mini-batch size and ρ(·) is the piecewise quadratic Huber function.

On labeled sequences, we additionally minimize a supervised cross-entropy loss based on the final-step prediction of each sequence:

(37) $$L_{\sup}=-\frac{1}{B}\sum_{i=1}^{B}\log p_{T_{i},y_{i}},$$

where $p_{T_{i},y_{i}}$ is the predicted probability of the ground-truth class $y_{i}$ at the terminal step $T_{i}$ of the *i*-th sequence.

The overall loss for the early-stopping diagnosis stage is

(38) $$L_{\mathrm{ESDRL}}=L_{\sup}+\omega_{\mathrm{RL}}L_{\mathrm{RL}},$$

where $\omega_{\mathrm{RL}}$ balances the supervised classification loss and the DRL loss. The validation split was used to choose $\omega_{\mathrm{RL}}=0.10$.

During inference, we adopt a greedy policy $a_{t}=\arg\max_{a}Q_{\theta_{q}}\left(s_{t},a\right)$, starting from the first window of each sequence. At each step, the agent decides whether to choose STOP or CONTINUE; the episode terminates once STOP is selected (i.e., an early stop is triggered) or when $T_{\max}$ windows have been acquired, thereby realizing an adaptive trade-off between diagnostic accuracy and measurement cost.

## 3. Experiment

### 3.1. Datasets and Difficulty Settings

In this work, we evaluate the proposed framework on two public bearing datasets: the University of Ottawa Rolling-element Dataset–Vibration and Acoustic Faults under Constant Load and Speed conditions (UORED-VAFCLS) and the Paderborn University (PU) bearing dataset. The two datasets are used to examine few-shot vibration diagnosis under limited labels and controlled measurement corruptions.

For UORED-VAFCLS and PU, the data split is performed at the raw-record level before window extraction. For each class, the training samples and the evaluation samples are generated from different raw CSV recordings. Sliding-window extraction is then conducted separately within the training-side and evaluation-side records. Therefore, the training and evaluation sets do not share the same raw recording or overlapping window segments.

The training-side records are used to sample the few-shot support set and to train the models. The evaluation-side records are used only to generate the Original, Easy, Medium, and Hard evaluation sets. The unlabeled healthy signals used for PK-SSL are taken from a separate healthy-only unlabeled pool; they are not used as labeled support samples and are not included in the evaluation-side records.

Given a raw recording of length $L_{\mathrm{raw}}$, the number of extracted base sequences is computed as

(39) $$N_{\mathrm{seq}}=\left\lfloor \frac{L_{\mathrm{raw}}-L}{S_{\mathrm{base}}}\right\rfloor +1,$$

where L=4096 is the base-sequence length and $S_{\mathrm{base}}$ is the stride used for base-sequence extraction. Each 4096-point base sequence is further divided into four non-overlapping 1024-point windows in the DRL formulation, as described in Section 3.2.1.

UORED-VAFCLS dataset (five-way classification). We use the accelerometer signal of UORED-VAFCLS [52] to construct a five-class bearing fault diagnosis task. The five classes are healthy condition, inner-race fault, outer-race fault, ball fault, and cage fault. The base-sequence length is L=4096, and the base-sequence stride is $S_{\mathrm{base}}=2048$. Under this setting, each evaluation-side source record produces 204 base sequences. For the K=6/8/10 settings, *K* labeled samples per class are sampled only from the training-side candidate sequences. In addition, 204 healthy base sequences from a separate healthy-only unlabeled pool are used only for PK-SSL pretraining. The data statistics are summarized in Table 3.

PU dataset (nine-way classification). The second benchmark is the PU bearing dataset, recorded on a dedicated test rig comprising an electric motor, a coupling shaft, the test bearing, and a load machine. We use the operating condition denoted as N09_M07_F10, which corresponds to a rotational speed of about 900 r/min (N09), a motor torque of 0.7 Nm (M07), and a radial load of 1000 N (F10). Vibration signals are measured by an accelerometer mounted on the housing of a 6203-type test bearing.

The PU task is a nine-way classification problem consisting of one healthy bearing and eight faulty bearings. The faulty bearings contain artificially induced outer-race and inner-race damages produced by EDM, electric engraving, and drilling. The per-class evaluation statistics are listed in Table 4.

Difficulty-level construction for evaluation. To make the controlled corruption protocol reproducible, Table 5 reports the parameter ranges used to generate the four difficulty levels: Original, Easy, Medium, and Hard. The Original setting uses the raw evaluation sequences without additional corruption. Easy, Medium, and Hard are generated by applying progressively stronger physically motivated perturbations to the evaluation-side sequences only. These perturbations emulate common measurement artifacts, including additive noise, impulsive interference, narrow-band attenuation, mild time warping, local signal loss, amplitude scaling, bias drift and circular shift.

The difficulty level is controlled by two factors: the perturbation budget and the perturbation strength. The perturbation budget refers to the number of corrupted measurement windows and the number of operations applied to each corrupted window, while the perturbation strength is determined by the SNR, impulse intensity, notch bandwidth, time-warp strength, masking ratio, amplitude scaling, bias drift, and circular-shift range. Easy applies one mild perturbation to each selected corrupted window, whereas Medium and Hard use larger budgets and stronger parameter ranges.

Although PhORJ uses related physically motivated transformations during few-shot training, the corrupted evaluation sequences are generated only from evaluation-side source records. They are not used for support-set sampling, training augmentation, PK-SSL pretraining, or model training.

**Figure 3:**
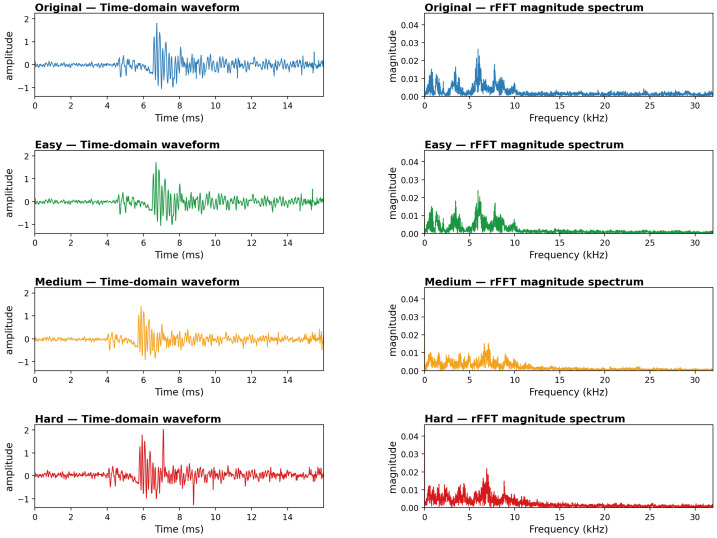
Representative examples of the four difficulty levels on the same PU bearing sample. Rows correspond to Original, Easy, Medium, and Hard, while the left and right columns show the time-domain waveform and rFFT magnitude spectrum, respectively. From top to bottom, the examples show progressively stronger measurement corruptions, including noise, local information loss, and spectral distortion.

As shown in Figure 3, the Original sample preserves the clean transient structure and concentrated spectral peaks. Easy introduces mild perturbations while keeping the main temporal and spectral patterns. Medium further weakens local structures and broadens spectral components. Hard shows stronger local distortion and a noisier spectrum. These examples show that the four difficulty levels correspond to progressively stronger controlled measurement corruptions.

### 3.2. Experimental Setup and Evaluation Metrics

#### 3.2.1. Windowing and Few-Shot Protocol

On both datasets, we adopt a sliding-window few-shot diagnosis setting. Given a raw vibration sequence $x\in R^{L}$, we first segment it into fixed-length windows with window length $L_{w}=1024$ and hop size $L_{s}=1024$, i.e., non-overlapping windows. This yields a sequence of windows ${\left\{x^{\left(t\right)}\right\}}_{t=1}^{T}$, where each window contains 1024 consecutive samples and corresponds to one measurement step.

In the DRL-based early-stopping diagnosis, the agent is allowed to acquire at most $T_{\max}=4$ windows for each sequence. At each step *t*, based on the current feature vector and confidence, the agent chooses between action CONTINUE (acquire the next window) and STOP (terminate and output the current prediction), thus trading off diagnostic accuracy against the number of acquired windows.

#### 3.2.2. Repeated Evaluation Protocol

For each *K*-shot setting, the labeled support set is fixed and shared by all compared methods. The support set contains exactly *K* labeled samples per class and is sampled only from the training-side candidate sequences.

To estimate the variability caused by evaluation-subset sampling, each method is evaluated on five fixed-seed evaluation subsets. The five sampling seeds are fixed before evaluation, and all compared methods are evaluated on exactly the same five subsets.

#### 3.2.3. Mislabeled Support Robustness Protocol

To evaluate robustness to imperfect annotations, we further construct mislabeled support sets on the PU dataset. After the clean *K*-shot support set is sampled, a proportion ρ of support samples is randomly selected, and its label is replaced by incorrect class labels uniformly sampled from the remaining classes. We evaluate ρ=0%, 10%, 20%. Only the labels in the support set are corrupted; the labels in the evaluation set remain unchanged. Under each *K*-shot setting and each label-noise rate, all compared methods use the same corrupted support set and the same evaluation subsets.

#### 3.2.4. Evaluation Metrics

We use classification metrics and a measurement–cost metric to evaluate diagnostic accuracy, class-wise performance, and measurement efficiency.

Classification accuracy. For each dataset and each difficulty level, we report the overall classification accuracy Acc∈[0,1]:

(40) $$\mathrm{Acc}=\frac{N_{\mathrm{correct}}}{N_{\mathrm{all}}},$$

where $N_{\mathrm{correct}}$ and $N_{\mathrm{all}}$ denote the number of correctly classified samples and the total number of evaluated samples, respectively.

Macro-averaged class-wise metrics. To describe the class-wise diagnostic performance, we further report Macro-Precision, Macro-Recall, Macro-F1, and Macro-AUC. For class *k*, let $TP_{k}$, $FP_{k}$, and $FN_{k}$ denote the true positives, false positives, and false negatives. The class-wise Precision, Recall, and F1-score are

(41) $${\mathrm{Precision}}_{k}=\frac{TP_{k}}{TP_{k}+FP_{k}},\;{\mathrm{Recall}}_{k}=\frac{TP_{k}}{TP_{k}+FN_{k}},$$

(42) $$F1_{k}=\frac{2\;{\mathrm{Precision}}_{k}\;{\mathrm{Recall}}_{k}}{{\mathrm{Precision}}_{k}+{\mathrm{Recall}}_{k}}.$$

The macro-averaged metrics are obtained by averaging the corresponding class-wise values over all *C* classes:

(43) $$\mathrm{Macro-Precision}=\frac{1}{C}\sum_{k=1}^{C}{\mathrm{Precision}}_{k},\;\mathrm{Macro-Recall}=\frac{1}{C}\sum_{k=1}^{C}{\mathrm{Recall}}_{k},$$

(44) $$\mathrm{Macro-F}1=\frac{1}{C}\sum_{k=1}^{C}F1_{k}.$$

Macro-AUC is computed using the one-vs-rest strategy based on the predicted class probabilities. Macro-averaged metrics are used because they assign equal weight to each fault class.

Overall cost *J*. To quantify the trade-off between diagnostic accuracy and measurement cost, we define the following cost function:

(45) $$J=c\;\bar{T}+\left(1-\mathrm{Acc}\right),$$

where $\bar{T}$ is the average number of measurement steps and c>0 is the metric cost coefficient. The metric coefficient is fixed to c=0.05 for all methods and all difficulty levels. This value treats one additional acquired window as equivalent to a five-percentage-point increase in classification error.

The first term $c\;\bar{T}$ measures the average measurement cost required to obtain a diagnosis, while the second term 1−Acc corresponds to the misclassification rate. Therefore, a smaller *J* indicates a better accuracy–measurement–cost trade-off. When comparing the proposed ESDRL with conventional fixed-window baselines, we report Acc, $\bar{T}$ and *J* simultaneously.

#### 3.2.5. Baseline Implementation and Fairness Protocol

Because the compared methods belong to different categories, including conventional CNN classifiers, metric-based few-shot methods, and DRL-based diagnosis agents, we adopt a unified fairness protocol to make the comparison transparent.

First, all methods use the same raw-record split and the same evaluation subsets. For each random seed and each *K*-shot setting, all compared methods use exactly the same labeled support set and the same evaluation samples.

Second, all supervised baselines are adapted to the few-shot setting using only the same *K* labeled samples per class. Conventional CNN baselines are trained from scratch on the same few-shot support set. Metric-based baselines construct prototypes from the same support samples. ProtoNet + PhORJ use the same support set with the same PhORJ augmentation budget. For CTQN, we set the evaluation horizon to $T_{\max}=4$ for comparability with the proposed method. However, $T_{\max}$ has a different operational meaning in CTQN: it denotes the number of windows used for fixed-horizon Q-value aggregation, rather than an adaptive STOP/CONTINUE stopping horizon. Therefore, CTQN is treated as a fixed-horizon sequential diagnosis baseline, while the proposed ESDRL learns an adaptive stopping policy whose actual stopping step can be 1, 2, 3, or 4.

Third, hyperparameters are selected only from the training/validation protocol and are fixed before evaluation. For comparable supervised-training hyperparameters, such as learning rate, batch size, and weight decay, we use comparable candidate ranges whenever the hyperparameter has the same meaning across methods. When a baseline has a recommended setting in the original paper, we include that setting as the default candidate and use the same selection rule. For the training budget, supervised classifiers are trained with the same maximum number of supervised epochs.

Fourth, we explicitly disclose method-specific information sources. PK-SSL uses a separate healthy-only unlabeled pool for self-supervised pretraining, but no fault labels are used in this stage. This unlabeled-data advantage is not hidden in the comparison; it is explicitly quantified through ablation variants such as no pretraining, generic SSL pretraining, and the full PK-SSL design. For augmentation fairness, PhORJ is applied only to labeled support samples, and the same augmentation budget is used whenever PhORJ is included.

#### 3.2.6. Training Details of ESDRL

To ensure reproducibility, we summarize the key implementation details of the Double-DQN training in Table 6. We use an experience replay buffer of capacity |D|=5000 transitions and start updating the Q-network after a warm-up of 2000 transitions. We train the Double-DQN agent for $N_{\mathrm{iter}}$ = 15,000 optimization iterations. At each iteration, we sample a batch of 16 trajectories, run the finite-horizon interaction (with at most $T_{\max}=4$ decision steps per trajectory), and then perform one Double-DQN update by uniformly sampling a mini-batch of size B=64 from the replay buffer. We adopt an ϵ-greedy exploration strategy. The discount factor is fixed to γ=0.9, and the DRL loss uses the Huber loss (smooth $\ell_{1}$). The target network is updated by soft updates with factor τ at each optimization step. All networks are optimized with Adam; we apply global gradient clipping with a maximum norm of 1.

The ESDRL-related coefficients are selected before the final evaluation. A small grid is used for $c_{\mathrm{step}}$, $\omega_{\mathrm{RL}}$, and γ, and the coefficient set is selected by minimizing the validation cost *J*. A sensitivity analysis around this selected setting is reported in Section 3.6.5.

Double-DQN training can be affected by exploration randomness, replay-buffer sampling, and target-network updates. To reduce training instability, ESDRL uses replay-buffer warm-up, Double-DQN target estimation, soft target-network updates, Huber loss, and global gradient clipping, as summarized in Table 6. These choices are used to stabilize the Q-value updates before the final stopping policy is evaluated.

#### 3.2.7. Convergence Behavior of ESDRL

To further examine the convergence behavior of the Double-DQN stopping module, we add a learning-curve comparison under the representative PU 8-shot setting. The compared supervised-only baseline uses the same PK-SSL initialization, PhORJ-augmented support set, and 1D backbone, but removes the Double-DQN stopping policy and is optimized only with cross-entropy loss. In contrast, ESDRL uses the same backbone and support set, but jointly optimizes the supervised classification loss and the DRL loss, and learns the STOP/CONTINUE policy.

The validation objective is

(46) $$J_{\mathrm{val}}=c{\bar{T}}_{\mathrm{val}}+\left(1-{\mathrm{Acc}}_{\mathrm{val}}\right),$$

where c=0.05. For the supervised-only baseline, fixed-window inference with T=4 is used. For ESDRL, ${\bar{T}}_{\mathrm{val}}$ is determined by the learned stopping policy.

**Figure 4:**
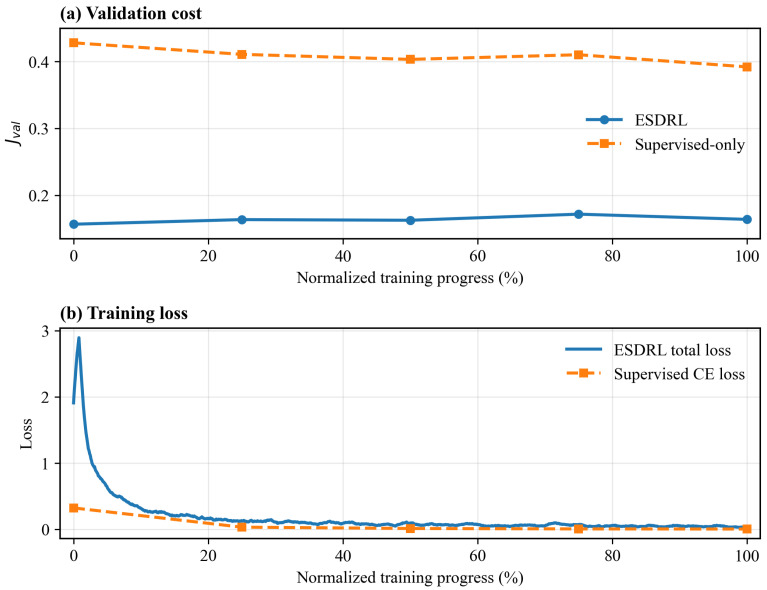
Convergence comparison between the supervised-only training loop and the proposed ESDRL under the representative PU 8-shot setting. (**a**) Validation cost $J_{\mathrm{val}}$. (**b**) Training loss. The supervised-only baseline removes the Double-DQN stopping policy and is trained only with cross-entropy loss. ESDRL uses the joint loss $L_{\mathrm{ESDRL}}=L_{\sup}+\omega_{\mathrm{RL}}L_{\mathrm{RL}}$. Lower $J_{\mathrm{val}}$ indicates a better validation accuracy–measurement–cost trade-off.

As shown in Figure 4a, ESDRL maintains a much lower validation cost than the supervised-only baseline during training. The ESDRL curve stays in a low and stable range, about 0.15–0.17, whereas the supervised-only baseline remains around 0.39–0.43. Figure 4b further shows that the ESDRL total loss decreases rapidly after the initial exploration stage and then approaches a stable range. The early peak is caused by the initial interaction and replay-buffer filling stage, after which the loss no longer shows persistent divergence. Together, these curves indicate that, under this representative setting, the Double-DQN stopping module reaches a stable accuracy–measurement–cost trade-off.

### 3.3. Comparison with Baseline Methods

We compare the proposed PK-SSL + PhORJ + ESDRL framework with representative baselines from three categories: conventional CNN classifiers, metric-based few-shot learning and DRL-based diagnosis. The compared methods include MSFFNET [53], MSCNN [54], WDCNN [55], MSAFCN [56], ProtoNet + PhORJ [57], and CTQN [51]. All methods follow the fairness protocol in Table 7.

Each main comparison is split into two paired tables. The first table reports Accuracy and Macro-AUC, and the second table reports Macro-Precision, Macro-Recall, and Macro-F1. Accuracy is reported as the mean ± standard deviation over five fixed-seed evaluation subsets, while Macro-Precision, Macro-Recall, Macro-F1, and Macro-AUC are reported as the corresponding mean values under the same evaluation protocol.

#### 3.3.1. Few-Shot Evaluation Under K=6/8/10

Table 8, Table 9, Table 10 and Table 11 summarize the few-shot classification results under all reported metrics. On UORED-VAFCLS, the Original, Easy, and Medium settings are close to saturation for several methods. For example, under the 8-shot Medium setting, WDCNN obtains 99.00±0.19% Accuracy and 100.00% Macro-AUC, while the proposed method obtains 99.19±0.38% Accuracy and 99.86% Macro-AUC. The difference between the strongest CNN baselines and the proposed method is therefore small in these easier settings.

The Hard setting gives a clearer comparison. Under the 8-shot Hard setting on UORED-VAFCLS, the proposed method obtains 95.84±0.93% Accuracy, 99.61% Macro-AUC and 95.73% Macro-F1. These values are close to WDCNN, which gives 95.62±0.55% Accuracy, 99.55% Macro-AUC and 95.72% Macro-F1. Compared with ProtoNet + PhORJ, however, the difference is larger: Accuracy increases from 85.84±1.24% to 95.84±0.93%, and Macro-F1 increases from 86.89% to 95.73%. At 10-shot Hard, the same comparison is 89.90±1.01%/90.47% for ProtoNet + PhORJ and 96.71±1.03%/96.80% for the proposed method. Thus, on UORED-VAFCLS, the proposed method is in the same performance range as the strongest CNN baselines and gives a clearer advantage over the metric-based few-shot baseline under stronger corruptions.

The PU dataset separates the methods more clearly. Under the representative PU-Hard 8-shot setting, the proposed method obtains 80.26±0.93% Accuracy, 95.10% Macro-AUC, and 82.23% Macro-F1. ProtoNet + PhORJ obtains 60.79±1.38% Accuracy, 89.15% Macro-AUC and 62.21% Macro-F1, while CTQN obtains 59.20±1.64% Accuracy, 90.46% Macro-AUC and 60.12% Macro-F1. The gains over ProtoNet + PhORJ are 19.47 percentage points in Accuracy and 20.02 percentage points in Macro-F1. The corresponding gains over CTQN are 21.06 and 22.11 percentage points. Macro-Precision and Macro-Recall show the same separation: the proposed method gives 82.52%/81.95%, compared with 63.70%/60.79% for ProtoNet + PhORJ and 60.86%/59.40% for CTQN. These results show that the improvement is also present in class-wise metrics, not only in the overall correct rate.

The shot-wise trend on PU also reflects the role of the proposed training pipeline under scarce labels. On PU-Hard, increasing *K* from 6 to 10 changes the proposed method from 79.43±1.46% to 80.80±1.40% Accuracy. In contrast, ProtoNet + PhORJ increases from 55.62±1.04% to 72.38±0.38%. The proposed method therefore already reaches a relatively high level at 6-shot, while the metric-based baseline depends more strongly on additional support samples.

#### 3.3.2. Robustness to Mislabeled Support Samples

Industrial deployment may involve a small number of incorrect few-shot labels. To examine this issue, we corrupt only the labels of the support samples and keep all evaluation labels unchanged. Table 12 and Table 13 report the robustness results under the representative 8-shot setting. The 0% column is the clean-support result under the same PU 8-shot protocol, while the 10% and 20% columns correspond to corrupted support labels.

Table 12 and Table 13 show the effect of support-label noise under the PU 8-shot setting. As expected, corrupting support labels reduces the performance of all methods. The degradation becomes more visible from Original to Hard, where the evaluation signals also contain stronger measurement corruptions.

Under the Hard setting with clean support labels, the proposed method obtains 80.26±0.93% Accuracy, 95.10% Macro-AUC, and 82.23% Macro-F1. ProtoNet + PhORJ obtains 60.79±1.38% Accuracy, 89.15% Macro-AUC and 62.21% Macro-F1, while CTQN obtains 59.20±1.64% Accuracy, 90.46% Macro-AUC and 60.12% Macro-F1. This indicates that the proposed pipeline has a higher starting point before label noise is introduced.

When 20% of the support labels are corrupted, the same Hard setting gives a clearer robustness comparison. The proposed method remains at 65.33±1.88% Accuracy, 89.98% Macro-AUC and 70.31% Macro-F1. In contrast, ProtoNet + PhORJ decreases to 40.53±1.71% Accuracy, 78.31% Macro-AUC and 42.25% Macro-F1, and CTQN decreases to 33.72±0.93% Accuracy, 77.12% Macro-AUC and 26.32% Macro-F1. WDCNN gives 45.26±1.01% Accuracy, 83.15% Macro-AUC and 45.58% Macro-F1. Thus, even when the support set contains incorrect annotations, the proposed method keeps a higher operating level in class decision accuracy.

The performance drop also shows different sensitivities to label noise. From 0% to 20% noise on PU-Hard, the proposed method decreases by 14.93 percentage points in Accuracy and 11.92 percentage points in Macro-F1. ProtoNet + PhORJ decreases by 20.26 and 19.96 percentage points, respectively, while CTQN decreases by 25.48 and 33.80 percentage points. WDCNN has a smaller absolute drop in Accuracy, but its final 20% noisy-label performance remains much lower than that of the proposed method. This comparison suggests that the advantage of the proposed framework comes from maintaining a stronger feature and decision baseline under noisy few-shot supervision, rather than from avoiding degradation entirely.

Overall, the baseline and mislabeled-support results give two observations. On UORED-VAFCLS, the proposed method reaches a performance level close to the strongest CNN baselines and is clearly better than ProtoNet + PhORJ under stronger corruptions. On PU, the gains are larger and remain visible across Accuracy, Macro-AUC and Macro-F1. The following section examines whether these classification results can be obtained with fewer acquired measurement windows.

### 3.4. Comparison with Fixed Measurement Length

This subsection examines whether the classification performance reported above can be obtained with fewer acquired measurement windows. We compare ESDRL with fixed-window inference strategies that always use a pre-defined number of windows $T_{\mathrm{fixed}}\in \left\{1,2,3,4\right\}$. For ESDRL, the maximum horizon is $T_{\max}=4$, but the actual stopping step is determined by the learned STOP/CONTINUE policy. The comparison is conducted on PU under the representative 8-shot setting.

The analysis uses three quantities: classification accuracy, the average number of acquired windows $\bar{T}$, and the evaluation cost $J=c\bar{T}+\left(1-\mathrm{Acc}\right)$ defined in Section 3.2.4. A smaller *J* means that the method reaches a better balance between diagnosis accuracy and measurement length.

**Figure 5:**
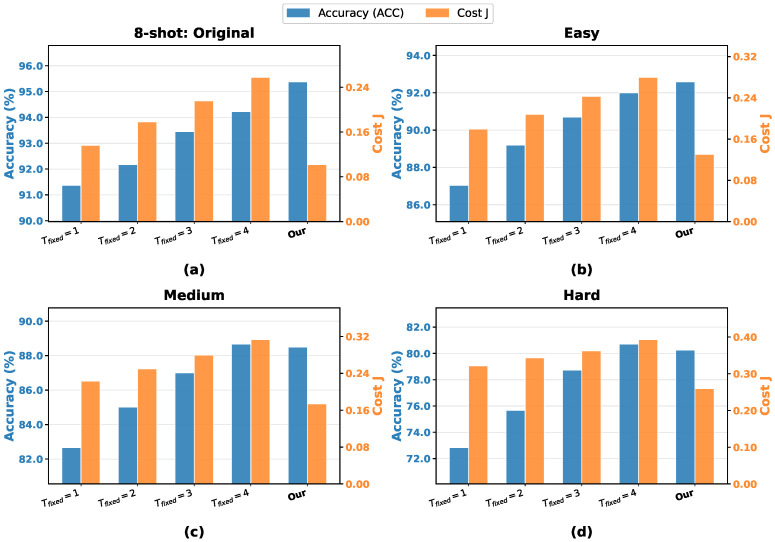
Accuracy–cost comparison between fixed-window inference and ESDRL on PU under the 8-shot setting. Subfigures (**a**–**d**) correspond to Original, Easy, Medium, and Hard, respectively. Blue bars show Accuracy, and orange bars show the evaluation cost *J*. ESDRL uses an adaptive stopping policy with $T_{\max}=4$, while the fixed-window baselines always use $T_{\mathrm{fixed}}=1,2,3,$ or 4 windows.

Figure 5 shows the basic trade-off. Increasing $T_{\mathrm{fixed}}$ generally improves accuracy, but it also increases *J* because every additional window adds measurement cost. Under the Original setting, fixed T=4 reaches 94.22% accuracy with J=0.2578, while ESDRL reaches 95.38% accuracy with only $\bar{T}=1.1132$ windows and J=0.1019. Under the Hard setting, fixed T=4 obtains 80.71% accuracy with J=0.3929, whereas ESDRL gives a similar accuracy of 80.26% with $\bar{T}=1.2432$ and a lower J=0.2596. Thus, ESDRL does not simply maximize accuracy by always reading all four windows; it keeps accuracy close to the long-window baseline while avoiding much of its measurement cost.

The same pattern is observed across the four difficulty levels. Compared with fixed T=4, ESDRL reduces *J* from 0.2578 to 0.1019 on Original, from 0.2800 to 0.1305 on Easy, from 0.3133 to 0.1738 on Medium, and from 0.3929 to 0.2596 on Hard. The relative reduction in *J* is therefore largest when the signal is easy, because most samples can be stopped after the first window, and remains substantial under Hard, where the policy still avoids unnecessary full-length inference for many samples.

#### 3.4.1. Sample-Level Stopping Behavior

**Figure 6:**
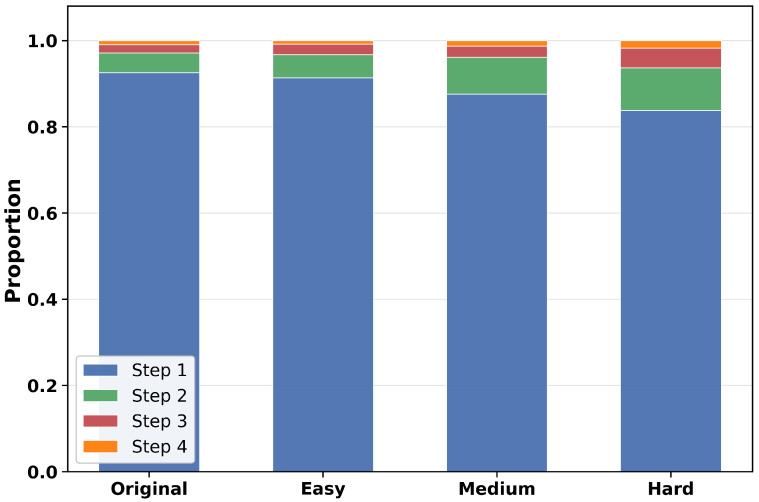
Stop-at-1/2/3/4 proportions of ESDRL on PU under the 8-shot setting. The four bars correspond to Original, Easy, Medium, and Hard. Harder conditions trigger more step-2/3/4 decisions, showing that the learned policy increases the measurement length when the input is less certain.

The fixed-window comparison reports only aggregate accuracy and cost. To examine how the policy behaves at the sample level, Figure 6 reports the proportions of samples stopped at steps 1, 2, 3 and 4 on PU under the 8-shot setting.

Most samples are still stopped at the first step, but the first-step proportion decreases as the corruption level increases. The stop-at-1 proportion is 92.54% on Original, 91.37% on Easy, 87.56% on Medium and 83.80% on Hard. Conversely, the proportion of samples using more than one window increases from 7.46% on Original to 16.20% on Hard. The proportion of step-3/4 decisions also increases from 2.90% on Original to 6.34% on Hard. This confirms that ESDRL is not equivalent to a fixed one-window shortcut; it acquires additional windows when the signal corruption becomes stronger.

**Figure 7:**
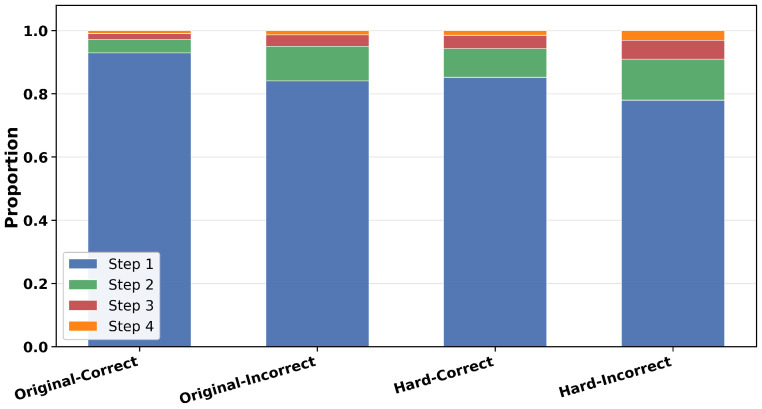
Stopping-step distributions for correctly and incorrectly classified samples on PU under the 8-shot setting. The bars compare Original-correct, Original-incorrect, Hard-correct, and Hard-incorrect samples. Incorrect samples have a larger proportion of later stopping steps, indicating that ambiguous samples tend to consume more measurement budget.

Figure 7 further separates correct and incorrect predictions. On the Original, 92.95% of correctly classified samples stop at step 1, while this proportion drops to 84.08% for incorrectly classified samples. On Hard, the corresponding proportions are 85.23% and 77.99%, respectively. Incorrect Hard samples also have the largest step-4 proportion, 3.05%, compared with 1.47% for correctly classified Hard samples. This behavior is reasonable: when the classifier remains uncertain, the policy tends to collect more evidence, although additional windows cannot always correct the decision.

Table 14 summarizes the same trend across shot settings. For every *K*, $\bar{T}$ increases from Original to Hard. For example, under 8-shot, $\bar{T}$ increases from 1.1132 to 1.2432. The lower-shot setting also requires slightly longer measurements: under Hard, $\bar{T}=1.2717$ for 6-shot, compared with 1.2289 for 10-shot. The policy therefore responds to both sources of uncertainty: stronger measurement corruption and fewer labeled support samples.

#### 3.4.2. Confidence-Threshold Stopping Baseline and Online Efficiency

We further compare ESDRL with a lightweight confidence-threshold stopping rule. At step *t*, the threshold baseline stops when the current prediction confidence exceeds a threshold:

(47) $$a_{t}=\left\{\begin{array}{cc} \mathrm{STOP}, & {\mathrm{conf}}_{t}\geq \tau ,\\ \mathrm{CONTINUE}, & \mathrm{otherwise}. \end{array}\right.$$

The threshold τ is selected from {0.80,0.85,0.90} using the validation criterion and is then fixed for evaluation. Table 15 compares fixed-window inference, the confidence-threshold baseline, and ESDRL on PU under the 8-shot setting.

Table 15 gives the quantitative comparison. The confidence-threshold rule is better than most fixed-window settings, confirming that confidence is a useful early-stopping signal. However, ESDRL gives a better accuracy–cost balance than the threshold rule on all four difficulty levels. For instance, on Medium, ESDRL improves accuracy from 85.92% to 88.50% and reduces *J* from 0.2030 to 0.1738. On Hard, the corresponding comparison is 80.26% versus 78.82% accuracy and 0.2596 versus 0.2773 in *J*.

Compared with fixed T=4, ESDRL reduces the average number of acquired windows from 4 to 1.1132–1.2432. The end-to-end latency is reduced from 6.360–6.760 ms to 1.848–1.976 ms per sample. On Original and Easy, ESDRL improves both Accuracy and *J*. On Medium and Hard, fixed T=4 is only slightly more accurate, with gains of 0.17 and 0.45 percentage points, respectively. However, its cost is much higher: *J* increases from 0.1738 to 0.3133 on Medium and from 0.2596 to 0.3929 on Hard. These results show that ESDRL favors shorter measurements when full four-window inference brings only a marginal accuracy gain.

#### 3.4.3. Static Computational Efficiency

In addition to online measurement efficiency, we report the static computational complexity of representative models. The latency is measured for a single 1024-point input window with batch size 1 on the same hardware platform. The same timing protocol is used for all methods.

Table 16 shows that the proposed model is not the lightest per window. Its per-window latency is 1.632 ms, compared with 0.435 ms for MSCNN and 1.049 ms for CTQN. Therefore, the efficiency gain of ESDRL does not come from a cheaper single forward pass. It comes from reducing the number of acquired windows. In Table 15, ESDRL usually uses about one to two windows, which keeps the end-to-end latency below 2 ms per sample despite the heavier backbone. This distinction is important: ESDRL improves online efficiency by adaptive measurement, not by using the smallest static model.

#### 3.4.4. Confidence Calibration Analysis

Because ESDRL uses confidence in the state representation and in the stopping reward, the confidence score should be informative enough to support stopping decisions. We therefore evaluate terminal confidence calibration under the representative PU 8-shot setting using reliability diagrams, expected calibration error (ECE), and Brier score. Lower ECE and Brier scores indicate better calibration.

**Figure 8:**
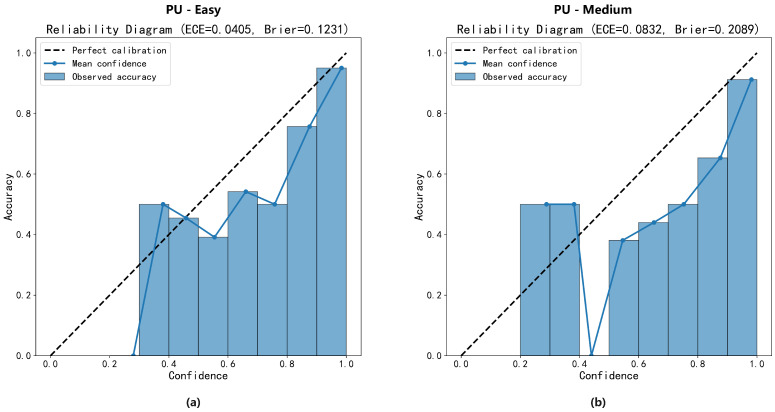
Reliability diagrams of terminal predictions on PU under the 8-shot setting. Subfigures (**a**,**b**) correspond to Easy and Medium, respectively. The dashed diagonal line denotes perfect calibration, the blue line shows mean confidence, and the bars show observed accuracy in each confidence bin.

Figure 8 and Table 17 show that confidence calibration becomes less ideal as the evaluation condition becomes harder. ECE increases from 0.0162 on Original to 0.1509 on Hard, and the Brier score increases from 0.0711 to 0.3534. This trend is expected because stronger corruptions produce more ambiguous predictions. Nevertheless, the reliability diagrams still show a positive relation between confidence and observed accuracy in the higher-confidence bins. The confidence score is therefore not perfectly calibrated, especially under Hard, but it remains informative for adaptive stopping.

Overall, the results in this subsection clarify the role of ESDRL. Fixed-window inference improves accuracy by always acquiring more windows, but this also increases measurement cost and latency. A confidence-threshold rule reduces unnecessary windows, but it uses a hand-crafted stopping boundary and is less consistent than the learned policy. ESDRL learns when additional windows are useful from the sequential confidence trajectory. It keeps the number of acquired windows close to one under easy conditions, increases the measurement length under harder conditions, and achieves the lowest evaluation cost *J* among the compared stopping strategies on PU 8-shot. These results support the use of adaptive early stopping as a measurement-efficient inference mechanism rather than merely as an additional classifier component.

### 3.5. Backbone Comparison

To examine whether the proposed 1D multi-scale attention backbone is a suitable shared feature extractor for the whole framework, we compare it with five representative 1D backbones: ResNet1D [58], Inception1D [59], ProtoCNN [60], AET [61], and CNN-Transformer [62]. The comparison is conducted on PU under the representative 8-shot setting. In this subsection, only the backbone architecture is changed. The PK-SSL pretraining, PhORJ augmentation, ESDRL training procedure, data split, support set, and hyperparameters are kept the same for all candidate backbones.

**Figure 9:**
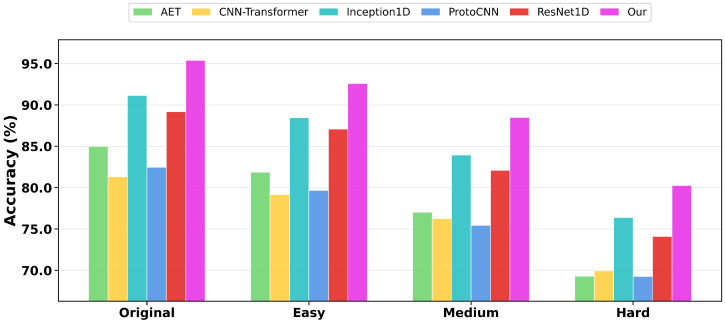
Backbone comparison on PU under the representative 8-shot setting. The x-axis groups the four evaluation difficulty levels: Original, Easy, Medium, and Hard. The y-axis reports classification Accuracy (%). All backbones are trained and evaluated using the same PK-SSL + PhORJ + ESDRL pipeline; only the backbone architecture is changed.

Figure 9 shows that the proposed backbone gives the highest accuracy under all four difficulty levels. On the Original setting, the proposed backbone reaches 95.38%, while the strongest alternative, Inception1D, reaches 91.14%. On Easy and Medium, the proposed backbone obtains 92.59% and 88.50%, compared with 88.46% and 83.95% for Inception1D. Under the Hard setting, the proposed backbone still remains the best, with 80.26% accuracy, whereas Inception1D and ResNet1D obtain 76.39% and 74.10%, respectively.

The margin over the strongest competing backbone is consistent but not exaggerated. Compared with Inception1D, the proposed backbone improves Accuracy by 4.24, 4.13, 4.55, and 3.87 percentage points on Original, Easy, Medium, and Hard, respectively. This is a meaningful difference because Inception1D already contains multi-scale convolutional branches and is therefore a strong comparator for vibration signals. The result indicates that the additional channel attention and the specific multi-scale depthwise design provide a stable benefit under the same downstream training pipeline.

The performance gap is larger when compared with backbones that are less tailored to short vibration windows. For example, under the Hard setting, the proposed backbone exceeds CNN-Transformer, ProtoCNN and AET by 10.33, 10.99 and 10.96 percentage points, respectively. These models can still learn useful representations, but their performance decreases more sharply when the evaluation signals contain stronger corruptions. This suggests that the proposed backbone is better matched to the local transient patterns and multi-scale temporal structures that appear in bearing vibration signals.

Overall, the backbone comparison supports using the proposed 1D multi-scale attention network as the shared encoder in PK-SSL, PhORJ, and ESDRL. The result indicates that the backbone itself contributes to a more stable representation under few-shot supervision and controlled measurement corruptions.

### 3.6. Ablation Studies

This subsection analyzes the contribution of the main components in the proposed framework. All ablation experiments are conducted on PU under the representative 8-shot setting, where the task is sufficiently challenging and the comparison is more informative. The analysis includes three levels: module-level ablation of PK-SSL, PhORJ, and ESDRL; fine-grained ablation of PK-SSL and PhORJ; and sensitivity analysis of the main ESDRL-related coefficients.

#### 3.6.1. Module-Level Ablation

**Figure 10:**
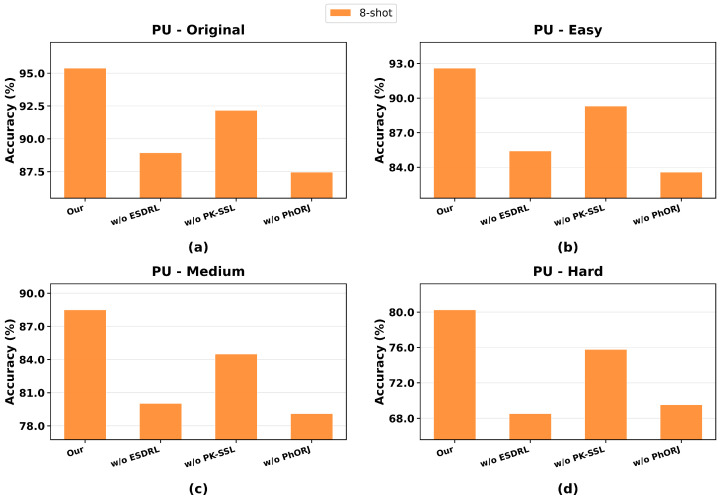
Module-level ablation on PU under the representative 8-shot setting. Subfigures (**a**–**d**) correspond to Original, Easy, Medium, and Hard, respectively. Each bar reports classification Accuracy (%). The comparison isolates the contributions of ESDRL, PK-SSL, and PhORJ under the same data split and training protocol.

Figure 10 reports the module-level ablation results. The four subfigures correspond to Original, Easy, Medium, and Hard, respectively. The compared variants are the full model, the model without ESDRL, the model without PK-SSL, and the model without PhORJ. In each variant, only the specified module is removed, while the remaining training pipeline is kept unchanged.

The full model gives the best result under all four difficulty levels, with 95.38%, 92.59%, 88.50%, and 80.26% accuracy from Original to Hard. Removing PK-SSL leads to a moderate but consistent decrease: the accuracy becomes 92.17%, 89.31%, 84.50%, and 75.78%. This shows that prior-guided pretraining mainly improves the feature initialization and becomes more useful when the evaluation signal is corrupted.

The drops caused by removing PhORJ or ESDRL are larger. Without PhORJ, the accuracy falls to 87.46%, 83.58%, 79.10%, and 69.52%, indicating that support-set augmentation is important for covering measurement variability under scarce labels. Without ESDRL, the accuracy becomes 88.94%, 85.42%, 80.02% and 68.52%, which is especially low under Hard. This suggests that adaptive stopping is not only an efficiency component; by using the confidence trajectory over sequential windows, it also improves the final decision under difficult samples.

The module-level results therefore show a complementary structure. PK-SSL improves the initial representation, PhORJ expands the limited support distribution, and ESDRL uses sequential evidence to decide when to stop. The largest degradation under Hard appears when ESDRL or PhORJ is removed, which is consistent with the goal of handling both scarce labels and corrupted measurements.

#### 3.6.2. Feature Visualization and Confusion Analysis

**Figure 11:**
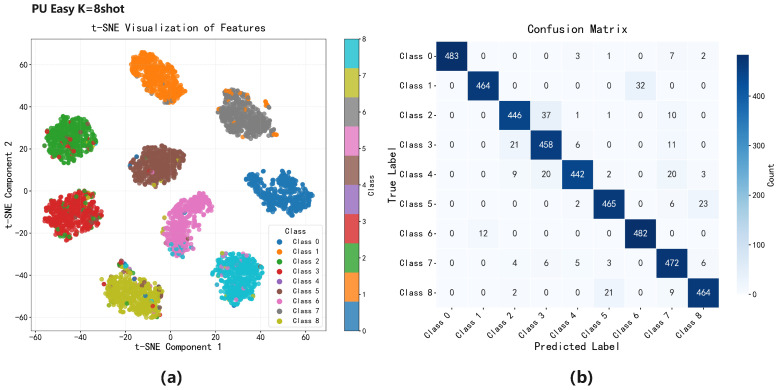
Feature visualization and confusion matrix on PU-Easy under the 8-shot setting. Subfigure (**a**) shows the t-SNE embedding of the learned features, where colors denote the nine PU classes. Subfigure (**b**) shows the confusion matrix, where diagonal entries denote correct predictions and off-diagonal entries denote misclassifications.

To further examine the learned representation, Figure 11 shows a representative visualization on PU-Easy under the 8-shot setting. The left subfigure gives the t-SNE embedding of the learned features, and the right subfigure gives the corresponding confusion matrix. This visualization is used as qualitative evidence only; the quantitative comparisons are reported in Table 8, Table 9, Table 10 and Table 11.

The t-SNE embedding in Figure 11a shows clear cluster structures for most classes, which is consistent with the high PU-Easy performance reported in Section 3.3. The confusion matrix in Figure 11b is strongly diagonal, but some residual confusions remain. For example, Class 1 is sometimes predicted as Class 6, and Class 2 and Class 3 also show mutual confusion. These errors indicate that several PU fault states still share similar local vibration patterns even after the proposed representation learning and augmentation pipeline.

#### 3.6.3. Fine-Grained Ablation of PK-SSL

The module-level ablation shows the effect of PK-SSL as a whole. To identify the source of this gain, Table 18 compares six pretraining strategies under the representative PU 8-shot setting: no pretraining, generic SSL pretraining, time-only PK-SSL, frequency-only PK-SSL, time + frequency pretraining without the consistency term, and full PK-SSL.

Table 18 shows that a single pseudo-label group does not produce the benefit of PK-SSL. Time-only and frequency-only pretraining both improve over no pretraining, and their combination gives a stronger result. Under Hard, no pretraining gives 75.78%, time-only PK-SSL gives 78.33%, frequency-only PK-SSL gives 77.64%, and the time + frequency variant without consistency gives 79.06%. Full PK-SSL further increases the Hard accuracy to 80.26%.

The consistency term gives a smaller but stable additional gain. Compared with time + frequency pretraining without consistency, full PK-SSL improves the accuracy by 0.76, 0.79, 1.09, and 1.20 percentage points from Original to Hard. This suggests that the main gain comes from prior-guided time- and frequency-domain supervision, while the consistency term acts as a regularizer that becomes more useful under stronger corruptions.

#### 3.6.4. Fine-Grained Ablation of PhORJ

We then examine the contribution of the additional physical perturbations in PhORJ. Table 19 compares classical ORJ, full PhORJ, and four removal variants. In each removal variant, one added perturbation type is removed while PK-SSL and ESDRL are kept unchanged.

Table 19 shows that full PhORJ consistently outperforms classical ORJ. The gains are 6.02, 6.41, 6.73 and 7.28 percentage points from Original to Hard. The increasing gain indicates that the added time–frequency perturbations are more useful when the evaluation signals contain stronger measurement corruptions.

Among the added perturbations, band-wise spectral operations are the most influential under the Medium and Hard settings. Removing them reduces the Hard accuracy from 80.26% to 74.81%, and the Medium accuracy from 88.50% to 83.27%. Removing local time masking also causes a clear decrease, especially under Medium and Hard. Phase jitter and global time warp produce smaller drops, but their removal is still weaker than the full design. These results show that PhORJ is not only a larger augmentation set; its frequency-band perturbations and local information-loss simulation provide the main robustness gain under stronger corruptions.

#### 3.6.5. Sensitivity of ESDRL-Related Coefficients

The main experiments use the validation-selected coefficient set $c_{\mathrm{step}}=0.05$, $\omega_{\mathrm{RL}}=0.10$, and γ=0.90. To examine whether the stopping behavior is sensitive to this choice, we vary one coefficient at a time under the representative PU 8-shot setting while keeping the other two fixed at their selected values. For each setting, Table 20 reports the average terminal accuracy, average stopping step $\bar{T}$, and average evaluation cost *J* over the four difficulty levels.

When sweeping $c_{\mathrm{step}}$, the evaluation cost *J* is always computed with the same metric coefficient c=0.05. Thus, $c_{\mathrm{step}}$ affects training and stopping behavior, while *c* in $J=c\bar{T}+\left(1-\mathrm{Acc}\right)$ remains fixed for fair comparison across rows.

The selected coefficient set gives the lowest average *J* among the tested values. For $c_{\mathrm{step}}$, the smallest value 0.01, allows longer measurements with $\bar{T}=1.2192$, but the average accuracy remains lower than the selected setting. Larger values do not consistently shorten the stopping length and also reduce the accuracy–cost balance. This indicates that an overly small or overly large continuation penalty is not optimal: the policy needs enough cost pressure to avoid unnecessary windows, but not so much that it stops before useful evidence is acquired.

For $\omega_{\mathrm{RL}}$, the selected value 0.10 gives the best balance. A smaller value weakens the stopping-policy learning, while larger values overemphasize the DRL objective relative to the supervised classification loss. For γ, 0.90 performs better than the shorter-horizon settings 0.50/0.70 and the setting 0.99. The value 0.99 increases $\bar{T}$ to 1.2065 without reducing *J*, suggesting that a very long-horizon preference encourages additional measurements without enough benefit in this finite four-step task.

In practical deployment, $c_{\mathrm{step}}$ can also be selected from engineering requirements rather than a large validation set. A latency-critical monitoring system can use a larger step cost to encourage earlier stopping, whereas a safety-critical machine can use a smaller step cost to collect more evidence before termination. Cost-driven settings can choose $c_{\mathrm{step}}$ according to the normalized cost of acquiring one additional vibration window relative to the cost of an incorrect diagnosis. Therefore, practical applications can adjust the step cost according to latency, acquisition cost, and fault-risk requirements.

Overall, the ablation studies support the internal logic of the framework. PK-SSL provides a useful prior-informed initialization, PhORJ improves robustness by expanding the support distribution with bounded time–frequency perturbations, and ESDRL converts the learned representation and confidence trajectory into adaptive stopping decisions. The components contribute through different mechanisms, and the strongest results are obtained when all three are used together.

## 4. Conclusions

In this paper, we studied measurement-efficient few-shot vibration fault diagnosis. The goal is not only to identify the fault class under scarce labels, but also to reduce the number of vibration windows required for a reliable decision. The proposed framework couples PK-SSL, PhORJ and ESDRL through a shared 1D backbone. PK-SSL provides prior-informed initialization from unlabeled healthy signals, PhORJ expands the scarce labeled support set with bounded physical perturbations, and ESDRL learns an adaptive STOP/CONTINUE policy for sequential window acquisition.

The experiments support three main findings within the tested setting. First, the proposed framework remains effective under representative few-shot settings with K=6/8/10 labeled samples per class on the UORED-VAFCLS and PU-bearing datasets. Second, under controlled measurement corruptions, PK-SSL and PhORJ improve representation quality and robustness, as shown by the module-level and fine-grained ablations. Third, ESDRL improves the accuracy–measurement–cost trade-off compared with fixed-window inference and a confidence-threshold stopping baseline. The stopping-step analysis further shows that the learned policy tends to acquire more windows for harder or more uncertain samples.

The current evidence should be interpreted within the scope of two public bearing datasets, fixed few-shot support sets, and controlled measurement corruptions. These results demonstrate robustness to the tested corruptions and measurement-efficient inference, but they do not by themselves establish full transfer to all rotating-machinery components or industrial systems. Future work will therefore extend the framework to other rotating components, including shafts and gears, and to more complex electromechanical systems such as electric machines. We will also examine broader operating-condition changes, multi-sensor monitoring, and deployment-oriented online diagnosis in real industrial environments.

## Figures and Tables

**Table 1:** Twelve time-domain prior indicators used in PK-SSL.

Index	Name	Equation
$T_{1}$	Root mean square (RMS)	$T_{1}=\sqrt{\frac{1}{N}\sum_{n=1}^{N}x{\left(n\right)}^{2}}$
$T_{2}$	Mean value	$T_{2}=\frac{1}{N}\sum_{n=1}^{N}x\left(n\right)$
$T_{3}$	Standard deviation	$T_{3}=\sqrt{\frac{1}{N}\sum_{n=1}^{N}{\left(x\left(n\right)-T_{2}\right)}^{2}}$
$T_{4}$	Skewness	$T_{4}=\frac{1}{N}\sum_{n=1}^{N}{\left(\frac{x\left(n\right)-T_{2}}{T_{3}}\right)}^{3}$
$T_{5}$	Kurtosis	$T_{5}=\frac{1}{N}\sum_{n=1}^{N}{\left(\frac{x\left(n\right)-T_{2}}{T_{3}}\right)}^{4}$
$T_{6}$	Crest factor	$T_{6}=\frac{\max_{n}\left|x\left(n\right)\right|}{T_{1}}$
$T_{7}$	Shape factor	$T_{7}=\frac{T_{1}}{\frac{1}{N}\sum_{n=1}^{N}\left|x\left(n\right)\right|}$
$T_{8}$	Impulse factor	$T_{8}=\frac{\max_{n}\left|x\left(n\right)\right|}{\frac{1}{N}\sum_{n=1}^{N}\left|x\left(n\right)\right|}$
$T_{9}$	Margin factor	$T_{9}=\frac{\max_{n}\left|x\left(n\right)\right|}{{\left(\frac{1}{N}\sum_{n=1}^{N}\sqrt{\left|x(n)\right|}\right)}^{2}}$
$T_{10}$	Zero-crossing rate	$T_{10}=\frac{1}{N-1}\sum_{n=1}^{N-1}1\;\left(x(n)x(n+1)<0\right)$
$T_{11}$	Peak-to-peak value	$T_{11}=\max_{n}x\left(n\right)-\min_{n}x\left(n\right)$
$T_{12}$	Absolute mean value	$T_{12}=\frac{1}{N}\sum_{n=1}^{N}\left|x\left(n\right)\right|$

**Table 2:** Twelve frequency-domain prior indicators used in PK-SSL.

Index	Name	Equation
$F_{1}$	Main-peak amplitude	$F_{1}=\max_{k}\left|X\left(k\right)\right|$
$F_{2}$	Main-peak frequency	$F_{2}=f_{k^{⋆}},\;k^{⋆}=\arg\max_{k}\left|X\left(k\right)\right|$
$F_{3}$	Spectral centroid	$F_{3}=\frac{\sum_{k}f_{k}\left|X\left(k\right)\right|}{\sum_{k}\left|X\left(k\right)\right|}$
$F_{4}$	Spectral spread (2nd central moment)	$F_{4}=\frac{\sum_{k}\left|X\left(k\right)\right|{\left(f_{k}-F_{3}\right)}^{2}}{\sum_{k}\left|X\left(k\right)\right|}$
$F_{5}$	Band energy 1 (lowest quarter)	$F_{5}=\frac{1}{K_{1}}\sum_{k\in B_{1}}\left|X\left(k\right)\right|$
$F_{6}$	Band energy 2	$F_{6}=\frac{1}{K_{2}}\sum_{k\in B_{2}}\left|X\left(k\right)\right|$
$F_{7}$	Band energy 3	$F_{7}=\frac{1}{K_{3}}\sum_{k\in B_{3}}\left|X\left(k\right)\right|$
$F_{8}$	Band energy 4 (highest quarter)	$F_{8}=\frac{1}{K_{4}}\sum_{k\in B_{4}}\left|X\left(k\right)\right|$
$F_{9}$	Spectral flatness	$F_{9}=\exp\;\left(\frac{1}{K}\sum_{k=1}^{K}\ln\left|X\left(k\right)\right|\right)$ $/\left(\frac{1}{K}\sum_{k=1}^{K}\left|X\left(k\right)\right|\right)$
$F_{10}$	85% roll-off frequency	$F_{10}=f_{k_{r}},\;\sum_{k\leq k_{r}}\left|X\left(k\right)\right|$ $\geq 0.85\sum_{k}\left|X\left(k\right)\right|$
$F_{11}$	Normalized spectral centroid	$F_{11}=\frac{F_{3}}{f_{\max}}$
$F_{12}$	High-frequency band energy	$F_{12}=\frac{1}{K_{h}}\sum_{k\in B_{\mathrm{high}}}\left|X\left(k\right)\right|$

**Table 3:** Data statistics of the UORED-VAFCLS dataset used in the five-class experiment. Base sequences are length-L=4096 segments extracted by a sliding window with stride $S_{\mathrm{base}}=2048$.

Class	Condition	Base Sequences
1	Healthy condition	204
2	Inner-race fault	204
3	Outer-race fault	204
4	Ball fault	204
5	Cage fault	204
–Unlabeled healthy base sequences (for PK-SSL only)		204

**Table 4:** Operating conditions and evaluation statistics of the PU dataset (nine-way classification). Base sequences are length-L=4096 segments extracted by a sliding window with stride $S_{\mathrm{base}}=2048$.

Class	Operating Condition	Base Sequences
1	KA01 (OR, EDM, level 1)	2470
2	KA03 (OR, Electric engraver, level 2)	2481
3	KA05 (OR, Electric engraver, level 1)	2472
4	KA07 (OR, Drilling, level 1)	2494
5	KA08 (OR, Drilling, level 2)	2471
6	KI01 (IR, EDM, level 1)	2470
7	KI03 (IR, Electric engraver, level 1)	2468
8	KI07 (IR, Electric engraver, level 2)	2481
9	K001 (healthy)	2470
–Unlabeled healthy base sequences (for PK-SSL only)		2480

**Table 5:** Parameter ranges used to construct the four difficulty levels. The complexity increases from Easy to Hard through a larger perturbation budget and stronger perturbation ranges.

Parameter	Original	Easy	Medium	Hard
Additional corruption	None	Yes	Yes	Yes
AWGN SNR (dB)	–	28–32	18–22	10–14
Impulse count per corrupted window	–	0–2	2–4	4–6
Impulse amplitude (×σ)	–	2.0–3.0	3.0–4.0	4.0–6.0
Notch bandwidth	–	0.02–0.05	0.05–0.10	0.08–0.15
Time-warp strength	–	0.00–0.02	0.02–0.05	0.05–0.08
Time-mask ratio	–	0.02–0.05	0.05–0.10	0.10–0.15
Amplitude scaling	–	0.90–1.10	0.85–1.15	0.80–1.20
Bias drift (×RMS)	–	0.05–0.10	0.10–0.20	0.20–0.30
Maximum circular shift (samples)	–	16–32	32–64	64–128
Operations per corrupted window	0	1	1–2	2–3
Narrow-band noise center	0.05–0.45, used only when narrow-band noise is selected			
Narrow-band noise bandwidth	0.01–0.05, used only when narrow-band noise is selected			
Narrow-band noise amplitude	0.2–0.6 × RMS, used only when narrow-band noise is selected			
Corruption templates	T1–T6 over four measurement steps; see note below			

*Note:* Each 4096-point sequence is divided into four non-overlapping 1024-point measurement steps. T1 corrupts step 1, T2 corrupts step 2, T3 corrupts step 3, T4 corrupts steps 1–2, T5 corrupts steps 3–4, and T6 corrupts alternating steps 1 and 3.

**Table 6:** Key hyperparameters for Double-DQN training in ESDRL.

Hyperparameter	Value	Unit/Meaning
Replay buffer capacity |D|	5000	transitions
Warm-up before updates	2000	transitions
Mini-batch size *B*	64	transitions/update
Maximum horizon $T_{\max}$	4	decision steps
Discount factor γ	0.90	dimensionless
DRL loss $L_{\mathrm{RL}}$	Huber	TD loss
Target-network update τ	0.005	soft-update factor
Exploration	ϵ-greedy	action exploration
Optimizer	Adam	optimizer type
Learning rate	$1\times 10^{-4}$	dimensionless
Gradient clipping	1.0	global norm
Step cost $c_{\mathrm{step}}$	0.05	reward cost/step
RL loss weight $\omega_{\mathrm{RL}}$	0.10	dimensionless

**Table 7:** Compact fairness protocol for baseline comparison. Under the same random seed and the same *K*-shot setting, all methods use the same raw-record split, the same *K*-shot support set, and the same evaluation subsets.

Method Group	Extra Training Information	Inference Protocol
CNN baselines	Same *K*-shot support set only; no PhORJ and no PK-SSL	Fixed-window classification
ProtoNet + PhORJ	Same support set with PhORJ augmentation under the same augmentation budget; no PK-SSL	Static prototype classification
CTQN	Same *K*-shot support set; no PhORJ and no PK-SSL	Fixed-horizon Q-value aggregation with $T_{\max}=4$
Ours	Same *K*-shot support set with PhORJ; PK-SSL uses a separate healthy-only unlabeled pool without fault labels	Adaptive STOP/CONTINUE stopping with maximum horizon $T_{\max}=4$

*Note:* CTQN uses $T_{\max}=4$ for fixed-horizon Q-value aggregation, whereas ESDRL uses $T_{\max}=4$ as the maximum stopping horizon and adaptively stops at step 1–4. PK-SSL is the only stage that uses healthy-only unlabeled sequences.

**Table 8:** Few-shot results on UORED-VAFCLS under K=6/8/10: Accuracy and AUC. Each cell reports Accuracy ± standard deviation/Macro-AUC (%).

Method	Diff.	6-Shot Acc./AUC	8-Shot Acc./AUC	10-Shot Acc./AUC
MSFFNET	Original	94.98±0.67/99.12	98.10±0.38/99.81	97.98±0.42/99.68
MSFFNET	Easy	94.98±0.67/99.12	97.98±0.43/99.80	97.98±0.42/99.69
MSFFNET	Medium	95.48±0.72/98.82	98.08±0.43/99.81	96.98±0.50/99.52
MSFFNET	Hard	90.14±1.10/96.79	89.30±0.68/98.22	88.80±0.62/96.82
MSCNN	Original	98.84±0.27/99.82	99.60±0.21/99.87	99.62±0.16/99.78
MSCNN	Easy	98.88±0.28/99.81	99.60±0.21/99.87	99.46±0.21/99.78
MSCNN	Medium	98.11±0.22/99.78	99.10±0.32/99.83	99.40±0.23/99.75
MSCNN	Hard	94.82±0.68/98.74	94.50±0.71/99.06	97.12±0.50/99.35
WDCNN	Original	99.30±0.30/100.00	99.30±0.30/100.00	99.90±0.13/100.00
WDCNN	Easy	99.42±0.22/100.00	99.42±0.22/100.00	99.90±0.13/100.00
WDCNN	Medium	99.00±0.19/100.00	99.00±0.19/100.00	99.74±0.17/100.00
WDCNN	Hard	95.52±0.55/99.26	95.62±0.55/99.55	96.20±0.39/99.86
MSAFCN	Original	99.48±0.10/100.00	99.66±0.06/100.00	99.86±0.10/100.00
MSAFCN	Easy	99.47±0.22/100.00	99.62±0.17/100.00	99.86±0.10/100.00
MSAFCN	Medium	98.76±0.19/99.96	98.86±0.23/99.96	98.80±0.31/99.89
MSAFCN	Hard	93.60±0.65/99.00	93.60±0.75/98.77	92.64±0.61/97.44
ProtoNet + PhORJ	Original	95.74±0.85/100.00	98.51±0.27/99.93	98.91±0.54/99.97
ProtoNet + PhORJ	Easy	96.04±0.65/100.00	98.61±0.29/99.94	99.01±0.66/99.97
ProtoNet + PhORJ	Medium	95.45±0.68/99.89	97.62±0.46/99.89	98.02±0.08/99.95
ProtoNet + PhORJ	Hard	84.95±1.08/97.64	85.84±1.24/97.97	89.90±1.01/98.74
CTQN	Original	98.22±0.32/99.98	98.94±0.32/99.99	99.10±0.21/100.00
CTQN	Easy	98.24±0.37/99.98	98.94±0.30/99.99	99.18±0.17/100.00
CTQN	Medium	98.24±0.37/99.96	98.88±0.30/99.99	98.98±0.17/99.98
CTQN	Hard	94.54±0.42/99.82	95.06±0.46/99.84	95.12±0.37/99.90
Ours	Original	99.50±0.05/100.00	99.68±0.13/99.95	100.00±0.00/100.00
Ours	Easy	99.47±0.05/100.00	99.68±0.13/99.95	100.00±0.00/100.00
Ours	Medium	99.13±0.17/100.00	99.19±0.38/99.86	99.80±0.88/99.95
Ours	Hard	95.54±1.00/99.27	95.84±0.93/99.61	96.71±1.03/99.26

**Table 9:** Few-shot results on UORED-VAFCLS under K=6/8/10: Macro-Precision, Macro-Recall and Macro-F1. Each cell reports Macro-Precision/Macro-Recall/Macro-F1 (%).

Method	Diff.	6-Shot P/R/F1	8-Shot P/R/F1	10-Shot P/R/F1
MSFFNET	Original	95.12/94.91/95.01	98.11/98.09/98.10	98.00/97.96/97.98
MSFFNET	Easy	95.12/94.91/95.01	97.99/97.97/97.98	98.00/97.96/97.98
MSFFNET	Medium	95.49/95.45/95.47	98.11/98.07/98.09	97.09/96.96/97.02
MSFFNET	Hard	90.29/90.08/90.18	91.59/89.26/90.41	91.59/88.79/90.17
MSCNN	Original	98.86/98.83/98.84	99.60/99.60/99.60	99.62/99.62/99.62
MSCNN	Easy	98.89/98.87/98.88	99.60/99.60/99.60	99.46/99.46/99.46
MSCNN	Medium	98.13/98.11/98.12	99.11/99.11/99.11	99.40/99.40/99.40
MSCNN	Hard	95.40/94.78/95.09	95.21/94.50/94.85	97.33/97.12/97.22
WDCNN	Original	99.32/99.31/99.31	99.32/99.31/99.31	99.90/99.90/99.90
WDCNN	Easy	99.43/99.43/99.43	99.43/99.43/99.43	99.90/99.90/99.90
WDCNN	Medium	99.03/99.00/99.01	99.03/99.00/99.01	99.75/99.73/99.74
WDCNN	Hard	95.85/95.60/95.72	95.85/95.60/95.72	96.23/96.20/96.21
MSAFCN	Original	99.48/99.49/99.48	99.66/99.65/99.65	99.86/99.86/99.86
MSAFCN	Easy	99.47/99.48/99.47	99.62/99.62/99.62	99.86/99.86/99.86
MSAFCN	Medium	98.77/98.75/98.76	98.88/98.85/98.86	98.84/98.80/98.82
MSAFCN	Hard	94.20/93.56/93.88	93.96/93.55/93.75	93.12/92.58/92.85
ProtoNet + PhORJ	Original	96.43/95.58/96.00	98.62/98.50/98.56	98.95/98.92/98.93
ProtoNet + PhORJ	Easy	96.64/95.89/96.26	98.70/98.61/98.65	99.04/99.01/99.02
ProtoNet + PhORJ	Medium	96.26/95.27/95.76	97.82/97.56/97.69	98.11/98.00/98.05
ProtoNet + PhORJ	Hard	87.59/84.87/86.21	87.99/85.82/86.89	91.16/89.80/90.47
CTQN	Original	98.31/98.23/98.27	98.97/98.95/98.96	99.14/99.11/99.12
CTQN	Easy	98.35/98.25/98.30	98.97/98.95/98.96	99.21/99.19/99.20
CTQN	Medium	97.98/97.85/97.91	98.89/98.88/98.88	99.01/98.98/98.99
CTQN	Hard	94.58/94.54/94.56	95.10/95.06/95.08	95.15/95.12/95.13
Ours	Original	99.50/99.48/99.49	99.69/99.69/99.69	100.00/100.00/100.00
Ours	Easy	99.48/99.47/99.47	99.69/99.68/99.68	100.00/100.00/100.00
Ours	Medium	99.13/99.11/99.12	99.22/99.17/99.19	99.81/99.80/99.80
Ours	Hard	95.86/95.76/95.81	95.63/95.83/95.73	96.93/96.67/96.80

**Table 10:** Few-shot results on the PU dataset under K=6/8/10: Accuracy and AUC. Each cell reports Accuracy ± standard deviation/Macro-AUC (%).

Method	Diff.	6-Shot Acc./AUC	8-Shot Acc./AUC	10-Shot Acc./AUC
MSFFNET	Original	45.42±2.19/84.11	47.04±1.04/84.36	49.36±1.04/86.37
MSFFNET	Easy	43.64±1.97/83.90	45.82±0.81/84.22	48.76±0.91/86.24
MSFFNET	Medium	43.98±1.76/83.67	45.88±1.01/83.76	48.24±1.16/86.06
MSFFNET	Hard	42.60±2.84/82.63	43.58±1.15/82.44	45.58±1.15/84.98
MSCNN	Original	59.04±1.36/92.53	58.90±0.91/92.53	60.92±0.74/93.38
MSCNN	Easy	56.72±0.52/91.89	57.84±1.03/92.30	59.24±0.61/93.12
MSCNN	Medium	56.30±0.52/91.30	57.80±1.21/92.03	56.90±1.56/92.25
MSCNN	Hard	52.54±1.72/89.59	53.18±0.64/90.36	53.80±1.42/90.30
WDCNN	Original	64.54±1.94/93.20	65.46±1.22/93.89	69.96±1.56/95.17
WDCNN	Easy	62.94±1.54/92.54	63.54±1.16/92.99	68.16±1.20/94.30
WDCNN	Medium	59.88±0.69/90.91	61.30±1.17/91.42	64.54±1.67/92.73
WDCNN	Hard	55.68±0.31/87.79	56.54±1.25/87.81	63.24±0.66/89.60
MSAFCN	Original	74.26±0.94/97.56	74.04±1.28/97.07	83.52±0.60/99.07
MSAFCN	Easy	72.08±0.52/96.66	72.18±1.02/96.31	81.58±0.69/98.41
MSAFCN	Medium	68.84±1.16/94.68	67.66±1.18/94.48	76.92±0.87/96.33
MSAFCN	Hard	62.40±1.61/90.54	62.38±0.30/90.68	69.84±0.46/92.28
ProtoNet + PhORJ	Original	69.80±1.18/95.55	78.19±1.57/97.43	89.96±0.35/99.24
ProtoNet + PhORJ	Easy	67.38±0.96/94.17	75.10±1.22/96.07	86.82±0.56/98.06
ProtoNet + PhORJ	Medium	62.70±0.88/91.48	69.56±1.24/93.77	81.44±0.55/96.78
ProtoNet + PhORJ	Hard	55.62±1.04/86.96	60.79±1.38/89.15	72.38±0.38/92.60
CTQN	Original	55.96±1.30/87.77	62.60±1.19/92.20	71.36±1.03/94.16
CTQN	Easy	56.48±1.37/87.96	62.84±0.68/92.02	70.46±0.99/93.98
CTQN	Medium	55.24±1.09/87.69	60.68±1.02/91.35	70.48±0.49/93.79
CTQN	Hard	53.28±1.36/86.58	59.20±1.64/90.46	66.90±1.20/92.68
Ours	Original	94.95±0.49/99.68	95.38±0.69/99.75	96.29±0.69/99.82
Ours	Easy	92.10±0.64/98.88	92.59±0.52/99.06	93.39±0.73/99.13
Ours	Medium	87.11±0.99/97.81	88.50±0.63/97.98	89.07±0.98/98.11
Ours	Hard	79.43±1.46/94.58	80.26±0.93/95.10	80.80±1.40/95.17

**Table 11:** Few-shot results on the PU dataset under K=6/8/10: Macro-Precision, Macro-Recall and Macro-F1. Each cell reports Macro-Precision/Macro-Recall/Macro-F1 (%).

Method	Diff.	6-Shot P/R/F1	8-Shot P/R/F1	10-Shot P/R/F1
MSFFNET	Original	46.12/45.37/45.74	46.76/46.90/46.83	49.44/49.18/49.31
MSFFNET	Easy	44.38/43.60/43.99	45.46/45.68/45.57	48.73/48.58/48.65
MSFFNET	Medium	44.36/43.95/44.15	45.69/45.75/45.72	48.32/48.09/48.20
MSFFNET	Hard	42.24/42.62/42.43	42.90/43.48/43.19	45.54/45.42/45.48
MSCNN	Original	60.15/58.85/59.49	59.88/57.85/58.85	63.23/60.84/62.01
MSCNN	Easy	58.91/56.30/57.58	59.37/57.71/58.53	60.93/59.49/60.20
MSCNN	Medium	57.03/56.06/56.54	60.54/57.74/59.11	57.68/56.73/57.20
MSCNN	Hard	53.16/52.16/52.66	55.19/53.05/54.10	55.11/53.92/54.51
WDCNN	Original	65.35/64.29/64.82	68.78/65.55/67.13	72.08/70.21/71.13
WDCNN	Easy	64.09/62.99/63.54	67.33/63.69/65.46	70.07/67.90/68.97
WDCNN	Medium	61.37/59.74/60.54	64.78/61.21/62.94	67.06/64.88/65.95
WDCNN	Hard	57.82/55.57/56.67	60.67/56.18/58.34	63.24/60.26/61.71
MSAFCN	Original	75.69/74.29/74.98	76.72/73.98/75.33	85.41/83.38/84.38
MSAFCN	Easy	73.36/72.10/72.72	74.97/72.14/73.53	83.44/81.46/82.44
MSAFCN	Medium	69.95/68.92/69.43	71.18/67.65/69.37	79.38/76.83/78.08
MSAFCN	Hard	63.61/62.50/63.05	66.59/62.37/64.41	72.92/69.78/71.32
ProtoNet + PhORJ	Original	72.88/69.79/71.30	79.40/78.19/78.79	90.28/89.96/90.12
ProtoNet + PhORJ	Easy	69.40/67.37/68.37	76.70/75.09/75.89	87.06/86.82/86.94
ProtoNet + PhORJ	Medium	62.18/62.69/62.43	71.30/69.56/70.42	81.83/81.44/81.63
ProtoNet + PhORJ	Hard	58.25/55.61/56.90	63.70/60.79/62.21	73.42/72.39/72.90
CTQN	Original	55.72/56.80/56.25	64.55/62.92/63.72	71.29/71.46/71.37
CTQN	Easy	55.34/56.30/55.82	64.50/63.11/63.80	70.45/70.52/70.48
CTQN	Medium	54.45/55.53/54.98	62.29/62.29/62.29	70.40/70.53/70.46
CTQN	Hard	52.54/53.61/53.07	60.86/59.40/60.12	66.79/66.93/66.86
Ours	Original	95.81/95.71/95.76	96.14/96.09/96.11	97.17/97.13/97.15
Ours	Easy	93.42/93.29/93.35	93.40/93.25/93.32	94.59/94.53/94.56
Ours	Medium	89.06/88.90/88.98	89.88/89.55/89.71	90.42/90.36/90.39
Ours	Hard	82.96/82.07/82.51	82.52/81.95/82.23	83.59/83.12/83.35

**Table 12:** Robustness to mislabeled support samples on PU under the 8-shot setting: Accuracy and AUC. Each cell reports Accuracy ± standard deviation/Macro-AUC (%).

Method	Diff.	0% Acc./AUC	10% Acc./AUC	20% Acc./AUC
WDCNN	Original	65.46±1.22/93.89	58.52±1.23/92.18	50.22±0.64/87.93
ProtoNet + PhORJ	Original	78.19±1.57/97.43	61.31±0.35/92.30	51.76±1.27/87.69
CTQN	Original	62.60±1.19/92.20	51.72±0.63/85.51	34.62±1.14/77.68
Ours	Original	95.38±0.69/99.75	87.24±1.24/98.99	80.45±1.98/97.15
WDCNN	Easy	63.54±1.16/92.99	57.82±1.04/91.68	50.80±1.35/87.62
ProtoNet + PhORJ	Easy	75.10±1.22/96.07	58.89±0.30/90.24	49.63±1.22/85.72
CTQN	Easy	62.84±0.68/92.02	51.98±0.64/85.59	34.94±1.27/77.63
Ours	Easy	92.59±0.52/99.06	84.69±1.04/98.01	77.75±1.68/95.66
WDCNN	Medium	61.30±1.17/91.42	56.82±0.91/90.15	48.02±0.39/85.48
ProtoNet + PhORJ	Medium	69.56±1.24/93.77	54.36±1.39/87.31	45.86±1.39/82.68
CTQN	Medium	60.68±1.02/91.35	51.80±0.34/85.33	35.04±1.26/77.70
Ours	Medium	88.50±0.63/97.98	79.99±1.73/96.49	72.78±1.97/93.74
WDCNN	Hard	56.54±1.25/87.81	52.22±1.60/87.26	45.26±1.01/83.15
ProtoNet + PhORJ	Hard	60.79±1.38/89.15	47.81±1.71/82.92	40.53±1.71/78.31
CTQN	Hard	59.20±1.64/90.46	52.26±0.71/85.04	33.72±0.93/77.12
Ours	Hard	80.26±0.93/95.10	72.38±1.39/92.84	65.33±1.88/89.98

**Table 13:** Robustness to mislabeled support samples on PU under the 8-shot setting: Macro-Precision, Macro-Recall and Macro-F1. Each cell reports Macro-Precision/Macro-Recall/Macro-F1 (%).

Method	Diff.	0% P/R/F1	10% P/R/F1	20% P/R/F1
WDCNN	Original	68.78/65.55/67.13	63.06/58.75/60.83	49.81/50.19/50.00
ProtoNet + PhORJ	Original	79.40/78.19/78.79	68.92/61.30/64.89	53.87/51.76/52.79
CTQN	Original	64.55/62.92/63.72	50.39/52.00/51.18	22.64/34.32/27.28
Ours	Original	96.14/96.09/96.11	90.65/90.34/90.49	84.74/82.59/83.65
WDCNN	Easy	67.33/63.69/65.46	62.08/57.29/59.59	50.58/50.57/50.57
ProtoNet + PhORJ	Easy	76.70/75.09/75.89	63.35/58.88/61.03	51.66/49.63/50.62
CTQN	Easy	64.50/63.11/63.80	50.69/52.28/51.47	22.51/34.63/27.28
Ours	Easy	93.40/93.25/93.32	88.12/87.77/87.94	82.20/79.83/81.00
WDCNN	Medium	64.78/61.21/62.94	60.96/56.62/58.71	48.33/48.26/48.29
ProtoNet + PhORJ	Medium	71.30/69.56/70.42	60.63/54.35/57.32	48.00/45.86/46.91
CTQN	Medium	62.29/62.29/62.29	50.32/52.07/51.18	22.60/34.71/27.38
Ours	Medium	89.88/89.55/89.71	83.19/82.79/82.99	78.88/75.70/77.26
WDCNN	Hard	60.67/56.18/58.34	56.40/52.13/54.18	45.82/45.34/45.58
ProtoNet + PhORJ	Hard	63.70/60.79/62.21	52.08/47.80/49.85	44.13/40.53/42.25
CTQN	Hard	60.86/59.40/60.12	51.37/52.45/51.90	21.71/33.41/26.32
Ours	Hard	82.52/81.95/82.23	74.88/74.20/74.54	72.01/68.69/70.31

**Table 14:** Average stopping step $\bar{T}$ of ESDRL on PU across shot settings and difficulty levels. Larger values indicate that the policy acquires more windows before stopping.

Shot Setting	Original	Easy	Medium	Hard
6-shot	1.1343	1.1692	1.2047	1.2717
8-shot	1.1132	1.1275	1.1762	1.2432
10-shot	1.1098	1.1260	1.1645	1.2289

**Table 15:** Online inference efficiency of different stopping strategies on PU under the 8-shot setting. All strategies use the same backbone and differ only in the stopping rule. Here $\bar{T}$ denotes the average number of acquired windows, and J↓ indicates that a lower *J* value is better.

Difficulty	Strategy	Acc. (%)	Avg. Windows $\bar{T}$	Latency/Sample (ms)	J↓
Original	Fixed T=1	91.37±0.38	1.000	1.685	0.1363
Original	Fixed T=2	92.17±0.85	2.000	3.352	0.1783
Original	Fixed T=3	93.45±0.76	3.000	5.125	0.2155
Original	Fixed T=4	94.22±0.40	4.000	6.360	0.2578
Original	Confidence threshold	93.60±0.63	1.155	1.820	0.1218
Original	ESDRL (Ours)	95.38±0.69	1.1132	1.848	0.1019
Easy	Fixed T=1	87.04±0.64	1.000	1.642	0.1796
Easy	Fixed T=2	89.19±0.50	2.000	3.344	0.2081
Easy	Fixed T=3	90.69±0.45	3.000	4.788	0.2431
Easy	Fixed T=4	92.00±0.67	4.000	6.717	0.2800
Easy	Confidence threshold	90.00±0.47	1.185	1.851	0.1593
Easy	ESDRL (Ours)	92.59±0.52	1.1275	1.949	0.1305
Medium	Fixed T=1	82.66±0.62	1.000	1.633	0.2234
Medium	Fixed T=2	85.01±0.63	2.000	3.392	0.2499
Medium	Fixed T=3	87.01±0.44	3.000	4.845	0.2799
Medium	Fixed T=4	88.67±0.88	4.000	6.700	0.3133
Medium	Confidence threshold	85.92±1.23	1.244	1.906	0.2030
Medium	ESDRL (Ours)	88.50±0.63	1.1762	1.933	0.1738
Hard	Fixed T=1	72.83±1.15	1.000	1.726	0.3217
Hard	Fixed T=2	75.68±1.29	2.000	3.436	0.3432
Hard	Fixed T=3	78.74±0.57	3.000	4.777	0.3626
Hard	Fixed T=4	80.71±0.74	4.000	6.760	0.3929
Hard	Confidence threshold	78.82±0.69	1.311	1.921	0.2773
Hard	ESDRL (Ours)	80.26±0.93	1.2432	1.976	0.2596

**Table 16:** Static computational complexity of representative methods for one 1024-point input window. Ours is heavier than MSCNN per window, but ESDRL usually evaluates far fewer windows per sample, as shown in Table 15.

Method	Params (M)	MACs (M)	Latency/Window (ms)	Throughput (Windows/s)
MSCNN	0.035	8.05	0.435	2299.8
CTQN	0.530	16.25	1.049	953.2
Ours	0.276	53.96	1.632	612.6

**Table 17:** Calibration metrics of terminal predictions on PU under the 8-shot setting. Lower ECE and Brier scores indicate better calibrated confidence.

Setting	ECE	Brier Score
PU-Original	0.0162	0.0711
PU-Easy	0.0405	0.1231
PU-Medium	0.0832	0.2089
PU-Hard	0.1509	0.3534

**Table 18:** Fine-grained ablation of PK-SSL on PU under the representative 8-shot setting. Results are reported as mean ± standard deviation.

Pretraining Strategy	Original	Easy	Medium	Hard
No pretraining	92.17±0.48	89.31±0.42	84.50±1.29	75.78±0.92
Generic SSL pretraining	92.63±0.59	90.12±0.60	85.49±0.67	76.31±0.94
Time-only PK-SSL	93.53±0.14	91.14±0.18	86.41±0.23	78.33±1.11
Frequency-only PK-SSL	94.09±0.85	91.12±0.66	85.75±0.98	77.64±0.44
Time + Freq w/o consistency	94.62±0.25	91.80±0.35	87.41±0.62	79.06±1.08
Full PK-SSL	95.38±0.69	92.59±0.52	88.50±0.63	80.26±0.93

**Table 19:** Fine-grained ablation of PhORJ on PU under the representative 8-shot setting. Results are reported as mean ± standard deviation.

Augmentation Variant	Original	Easy	Medium	Hard
Classical ORJ	89.36±0.90	86.18±0.94	81.77±1.08	72.98±1.00
PhORJ w/o band ops	92.60±1.08	89.57±0.98	83.27±1.25	74.81±1.51
PhORJ w/o phase jitter	94.10±0.35	92.16±0.52	87.00±0.44	79.50±0.87
PhORJ w/o global time warp	94.77±0.67	92.54±0.50	87.20±0.68	79.06±1.67
PhORJ w/o local time masking	92.26±0.78	90.88±1.17	85.72±1.09	77.60±1.05
Full PhORJ	95.38±0.69	92.59±0.52	88.50±0.63	80.26±0.93

**Table 20:** Compact sensitivity summary of the main ESDRL-related coefficients on PU under the representative 8-shot setting. For each parameter value, we report the average accuracy, average stopping step $\bar{T}$, and average cost *J* over the four difficulty levels.

Parameter	Value	Avg. Acc. (%)	Avg. Step $\bar{T}$	Avg. *J*
$c_{\mathrm{step}}$	0.01	88.37	1.2192	0.1773
	0.03	88.61	1.1735	0.1726
	0.05	89.18	1.1650	0.1664
	0.07	88.14	1.1978	0.1785
	0.10	88.41	1.1853	0.1751
$\omega_{\mathrm{RL}}$	0.05	88.19	1.1808	0.1771
	0.10	89.18	1.1650	0.1664
	0.20	88.35	1.1803	0.1755
	0.50	88.04	1.2240	0.1808
γ	0.50	88.58	1.1727	0.1729
	0.70	88.63	1.1717	0.1723
	0.90	89.18	1.1650	0.1664
	0.99	88.48	1.2065	0.1755

## Data Availability

The raw bearing vibration datasets analyzed in this study are publicly available from their original data providers: the University of Ottawa UORED-VAFCLS dataset and the Paderborn University bearing dataset.
